# Technical aspects of inter-recti distance measurement with ultrasonographic imaging for physiotherapy purposes: the scoping review

**DOI:** 10.1186/s13244-023-01443-4

**Published:** 2023-05-18

**Authors:** Agnieszka Opala-Berdzik, Magdalena Rudek-Zeprzałka, Justyna Niesporek, Maciej Cebula, Jan Baron, Katarzyna Gruszczyńska, Augusto Gil Pascoal, Patrícia Mota, Daria Chmielewska

**Affiliations:** 1 Institute of Physiotherapy and Health Sciences, Department of Physiotherapy in Internal Diseases, The Jerzy Kukuczka Academy of Physical Education, Katowice, Poland; 2 Department of Physical Medicine, The Jerzy Kukuczka Academy of Physical Education, Katowice, Poland; 3 Department of Radiology and Nuclear Medicine, Medical University of Silesia, Katowice, Poland; 4 Individual Specialist Medical Practice Maciej Cebula, Katowice, Poland; 5 Faculty of Human Kinetics, Interdisciplinary Centre of Human Performance (CIPER), Biomechanics and Functional Morphology Laboratory (LBMF), University of Lisbon, Lisbon, Portugal; 6 H&TRC – Centro de Investigação em Saúde e Tecnologia, Escola Superior de Tecnologia da Saúde de Lisboa (ESTeSL) – Instituto Politécnico de Lisboa, Lisbon, Portugal; 7 Institute of Physiotherapy and Health Sciences, Electromyography and Pelvic Floor Muscles Assessment Laboratory, Department of Physical Medicine, The Jerzy Kukuczka Academy of Physical Education, Katowice, Poland

**Keywords:** Linea alba, Inter-recti distance, Diastasis recti abdominis, Ultrasonographic imaging, Measurement procedure

## Abstract

**Background:**

Inter-recti distance (IRD) measurement using musculoskeletal USI has been used in physiotherapy research, in particular, to investigate pregnancy-related diastasis recti abdominis (DRA) and to seek its effective treatment methods. Severe and untreated diastasis may result in the formation of umbilical or epigastric hernias.

**Objective:**

This study aimed to systematically map physiotherapy-related research articles that included descriptions of IRD measurement procedures using USI to present their similarities and differences, and formulate recommendations on the procedure.

**Design:**

A scoping review was conducted according to PRISMA-ScR guidelines, including 49 of 511 publications from three major databases. Publications were selected and screened by two independent reviewers whose decisions were consulted with a third reviewer. The main synthesized data items were: the examinees’ body position, breathing phase, measurement sites, and DRA screening methods. The final conclusions and recommendations were the result of a consensus between seven reviewers from four research centers.

**Results:**

Studies used 1–5 measurement sites that were differently determined. IRD was measured at the umbilicus (*n* = 3), at its superior (*n* = 16) and/or inferior border (*n* = 9), and at different levels: between 2 and 12 cm above the umbilicus, or a third of the distance and halfway between the umbilicus and xiphoid (*n* = 37); between 2 and 4.5 cm below the umbilicus or halfway between the umbilicus and pubis (*n* = 27). Different approaches were used to screen subjects for DRA.

**Conclusions:**

The discrepancies between the measurement procedures prevent between-study comparisons. The DRA screening method should be standardized. IRD measurement protocol standardization has been proposed.

**Critical relevance statement:**

This scoping review indicates that the inter-recti distance measurement procedures using ultrasound imaging differ between studies, preventing between-study comparisons. Based on the results synthesis, the measurement protocol standardization has been proposed.

**Key points:**

- The inter-recti distance measurement procedures using USI differ between studies.
- Proposed standardization concerns body position, breathing phase, measurements number per location.
- Determination of measurement locations considering individual linea alba length is suggested.
- Recommended locations: umbilical top, ½ of umbilical top-xiphoid, ¼ of umbilical top-xiphoid/pubis distances.
- Diastasis recti abdominis diagnostic criteria are needed for proposed measurement locations.

**Graphical Abstract:**

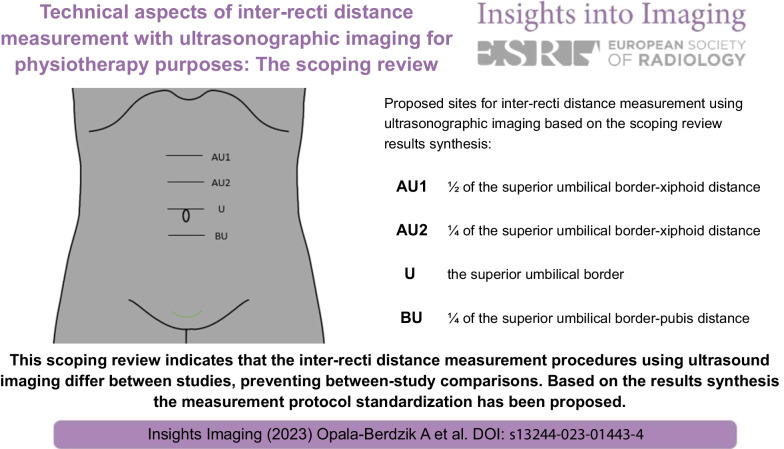

**Supplementary Information:**

The online version contains supplementary material available at 10.1186/s13244-023-01443-4.

## Introduction

Musculoskeletal ultrasonographic imaging (USI) has become a valuable tool in physiotherapy research and practice [1–5]. Among others, it has been used to measure the width of the fibrous linea alba, also called the inter-recti distance (IRD) [6, 7]. The most common purpose of this measurement is to investigate pregnancy-related diastasis recti abdominis (DRA) or monitor the impact of specific exercises on this condition [8–13]. DRA mostly develops in advanced pregnancy and manifests itself as an overstretched linea alba leading to the excessive separation of the recti abdominis muscles. In approximately 40% of women, the condition persists after pregnancy [14, 15] showing increased linea alba laxity [8, 10] that is accompanied by decreased thickness, strength, and impaired function of the abdominal muscles [16–18]. Due to its appearance, persistent DRA negatively impacts women’s self-esteem and body image [19, 20]. If pronounced, the condition may result in insufficient soft tissue protection of the uterus in successive pregnancies and the formation of umbilical/epigastric hernia that may require surgical intervention [21–24]. Possible pregnancy-related DRA risk factors and the association of post-pregnancy DRA with chronic low back pain or support-related pelvic floor dysfunction are still under investigation [25–30]. According to the systematic reviews, DRA may be associated with impaired abdominal muscle strength [31], health-related quality of life [31, 32], body image satisfaction, abdominal pain [32], low back pain severity [31], and pelvic organ prolapse [32]. No significant association was observed between the DRA and lumbopelvic pain or urinary incontinence [31, 32]. However, further investigation is needed to verify these relationships because of the weak methodological quality of the existing studies and their heterogenicity regarding study design and population [31, 32]. Numerous studies have investigated the impact of specific therapeutic exercises or other physiotherapy methods on pregnancy-related DRA [8, 33–37]. Physiotherapy has been shown to improve the condition [38–40]. However, according to a recent systematic review and meta-analysis, there is currently no high-quality scientific evidence on the most effective exercise programs in the treatment of postpartum DRA [41]. Besides research on pregnancy-related DRA, IRD measurements have also been performed in studies on athletes [42, 43] and individuals with tendinopathy, dysmenorrhea, or lumbopelvic pain [44–46].

In physiotherapy research involving IRD measurement, musculoskeletal USI has been widely used owing to its high reliability [7, 18, 47–50]. However, review studies have indicated that IRD measurement procedure has not been standardized [39, 40]. The main discrepancies between the study protocols concern measurement sites (supraumbilical and infraumbilical) and diagnostic criteria for DRA [14, 38–40]. They also apply to the examinee’s body position, way of abdominal muscle activation, or breathing phase during image capturing. Differences in the measurement procedure may impact study outcomes and make the comparison of findings obtained by different research centers difficult or impossible [40]. Therefore, systematic mapping of physiotherapy-related studies involving IRD measurement with USI and discussions on specific aspects of the measurement procedures in the light of existing knowledge on musculoskeletal USI are warranted. All these should help formulate recommendations on best practices in performing and describing IRD assessment methods. If future studies follow such recommendations and use standardized measurement procedures, reports on DRA incidence or treatment effects could be compared. The recommendations may also help physicians and physiotherapists make more accurate DRA diagnoses and decide what conditions might benefit from physiotherapy. This, in turn, may prevent adverse effects of untreated DRA.

Therefore, this scoping review aimed to collect all peer-reviewed publications related to physiotherapy and physical exercise/training that describe IRD measurement procedures using USI. Based on discussions on similarities and differences of the procedures, and the completeness of their descriptions, it was aimed to formulate recommendations on the IRD measurement protocol standardization, to be considered in designing future physiotherapy studies and used in physiotherapy practice.

## Methods

This scoping review was designed and conducted according to the Preferred Reporting Items for Systematic Reviews and Meta-Analyses, Extension for Scoping Reviews (PRISMA-ScR) [51, 52]. The PRISMA-ScR checklist is provided in Additional file 1: S1.

### Protocol

The study protocol was jointly developed a priori by two review leads (A.O.B. and D.C.). It was registered at the International Platform of Registered Systematic Review and Meta-analysis Protocols (INPLASY). The registration number is 202290116.

### Eligibility criteria

The eligibility criteria were developed by two reviewers (A.O.B. and D.C.) with experience in research and practice on women’s health physiotherapy and IRD assessment using USI. Articles published in English starting with the earliest available until the last database entry were considered for review. To assure high-standard measurement procedure descriptions, the included publications were peer-reviewed research papers or registered study protocols. Table 1 presents detailed inclusion and exclusion criteria.

**Table 1:** Inclusion and exclusion criteria used in the two-phase selection process to identify publications describing the measurement of inter-recti distance (IRD)/diastasis recti abdominis (DRA) with ultrasonographic imaging (USI)

	Inclusion	Exclusion
Publication type	Full-text of a peer-reviewed original research article or registered study protocol	Only abstract form, conference proceeding, letter, review
*Phase I—title and abstract*		
Population	Human adults	Non-humans and human infants, children, and adolescents
Concept	IRD/linea alba width/DRA measurement procedure with USI (detailed procedure description)	USI or other procedures not related to IRD/linea alba width/DRA measurement; IRD/linea alba width/DRA measurement with other tools, e.g., manual caliper, tape measure, ruler, magnetic resonance, computer tomography, palpation, and intraoperatively
*Phase II—full text*		
Context*	Field of physiotherapy or physical exercise/training	Field of surgery (plastic, general) or other medical fields not related to physiotherapy or physical exercise/training

*Selection based on the authors’ affiliation, study aims, and information provided in the main text of the publication

### Information sources

To identify potentially relevant publications, the PubMed, Embase (Elsevier), and Ovid (Medline) bibliographic databases were searched by two independent reviewers (D.C. and A.O.B.). The most recent electronic literature search was conducted on August 31, 2022. The search was supplemented by hand searching and scanning the reference lists of the included or relevant sources of evidence.

### Search strategy

The search strategy was developed by two reviewers (D.C. and A.O.B.) who gained their knowledge through video tutorials and closely cooperated with an experienced librarian from the Medical University Library. For PubMed, the final search strategy used was as follows: ((interrect*[tiab]) OR (inter-rect*[tiab]) OR (linea alba[tw]) OR (recti abdominis[tiab]) OR (recti muscle*[tiab]) OR (rectus muscle[tw]) OR (rectus abdom*[tiab]) OR (recti[tiab]) OR (rectus abdominis[mh])) AND ((diastasis[tiab]) OR (separation[tw]) OR (width[tw]) OR (distance[tiab]) OR (widening[tw])) AND ((USI[tw]) OR (ultrasound imaging[tiab]) OR (ultrasonography[tiab]) OR (ultrasonography[mh]) OR (US[tw]) OR (ultrasound[tiab])); filter: English.

All search results were saved, and duplicate publications were removed manually. To reduce the risk of error, two researchers (D.C. and M.R.Z.) independently removed duplicates and compared the outcomes of their selection.

### Selection of sources of evidence

Two standardized forms were developed (using the Microsoft Forms software, Office 365) to guide the screening of the articles identified through the search strategy. Forms I and II concerned title and abstract, and full-text screening, respectively (Additional file 1: S2 and S3). These forms, along with the explanation and elaboration documents (Additional file 1: S4), were developed and refined by two review leads (D.C. and A.O.B.) during the prescreening of 50 random publications. The prescreening was a continuous interactive process of refining the questions to improve their appropriateness, accuracy, and comprehensiveness and ensure that filling out the forms will enable capturing all relevant publications. After both forms had been prepared, four reviewers (D.C., A.O.B., M.R.Z, and J.N.) performed calibration exercises, independently screening a sample of 30 titles and abstracts and filling out Forms I. The Kappa coefficient showed an interrater agreement of 0.80–0.93. The discrepancies between the reviewers’ answers and any unclear issues were discussed until a consensus was reached. Minor refinements were then made to Form I and to the explanation and elaboration document. As the interrater agreements were satisfactory [52], the screening of the titles and abstracts of all remaining publications was continued independently by two reviewers (D.C./J.N. and M.R.Z.). After completing Forms I for all publications, the reviewers compared the answers, discussed and resolved any disagreements by consensus. In cases of uncertainty, an additional reviewer (A.O.B.) was consulted to make the final decision.

The same steps were followed for full-text screening and filling out Forms II. In this phase, the calibration exercises concerned screening a sample of 20 full texts. The interrater agreements were excellent (Kappa coefficient = 1).

### Data charting process

A Data-charting Form was jointly developed by two review leads (A.O.B. and D.C.) to extract relevant information on the IRD measurement procedure from the included sources of evidence (Additional file 1: S5). Two reviewers (M.R.Z./J.N. and A.O.B./D.C.) independently copied appropriate extracts from full texts and pasted them into separate forms. To facilitate comparisons, extracts by both reviewers were pasted in two adjacent columns of the final version of the Data-charting Form.

### Data items

The following data items on the IRD measurement procedure using USI were sought: publication characteristics (first author, publication year, country/countries of the research center, main study objective); population characteristics (sex, age, body mass index [BMI; mean ± standard deviation], DRA presence, and for females, the number of pregnancies/deliveries, mode of delivery); specific aspects of the measurement procedure (examinee’s body position during the assessment at rest, muscle activation/task during the examination, the respiratory phase/pattern during image capturing (Table 2), the examiner’s profession and experience (hours of training and years of practice), ultrasonographic scanner (brand, type, mode, and field of view) and transducer (type and frequency), measurement site/sites; cutoff values for “normal” IRD or DRA (i.e., IRD considered normal or indicative of a pathological condition), number of images taken at each measurement site and whether measurements were averaged, image processing and measurement methods (online or offline); Table 3).

**Table 2:** Study objectives, participants, and conditions for performing the inter-recti distance (IRD) measurement using ultrasonographic imaging (USI) in 49 study protocols

	Study objectives	Participants	Body position during assessment at rest*	Assessment during specific muscle activity/task	Breathing
Abuín-Porras 2019 (Spain) [42]	To compare abdominal muscle thickness and IRD between female rugby players and non-athletes	32 nulliparas: 16 rugby players, mean age: 24.7 ± 4.9 yr, BMI: 23.4 (21.9–24.7); 16 non-athletes, mean age: 27.9 ± 6.1 yr, BMI: 22.8 (20.5–25.7)	Supine	Not applicable	At the end of expiration
Arranz-Martín 2022 (Spain/Canada) [74]	To determine the acute effect of an abdominal hypopressive exercise (AHE) on IRD, linea alba distortion, and abdominal muscle thickness among primiparous women and to compare this effect with that of other abdominal exercises	46 primiparas at 3mo after singleton vaginal delivery, mean age: 35 ± 4 yr; median BMI: 22.7 (interquartile range: 21.1–25.8)	Hook lying	- AHE: trunk axially extended, wrists, ankles dorsiflexed, shoulders medially rotated; after one breathing cycle, in apnea, ribs expanded laterally, abdominal wall moved inward and cranially - ADIM: after one breathing cycle, navel drawn in toward the spine - Semi curl-up: after breathing cycle, head and shoulders off the table, arms at sides, inferior scapular angles in contact with the table - ADIM + semi curl-up	Pattern/phase related to specific exercise
Balasch-Bernat 2021 (Spain) [56]	To evaluate the differences in IRD and abdominopelvic function between nulliparous, primiparous and multiparous women	75 women: 25 primiparas and 25 multiparas at 6mo after vaginal delivery, and 25 nulliparas; mean age: 34.2 ± 2.6, 35.0 ± 4.0, 33.2 ± 4.6 yr, respectively; BMI: 23.4 ± 4.2, 22.3 ± 3.0, 22.3 ± 2.9, respectively	Supine, slight knee and hip flexion, a pillow under the knees, arms along the body	- ADIM: after inhaling and exhaling, navel drawn in toward the spine - Curl-up: head and shoulders lifted, lower scapular angles off the examination table	Not specified
Barbosa 2013 (Brazil) [60]	To investigate the accuracy of IRD measurement using a caliper compared with USI	106 paras 72 h after delivery (62.2% cesarean, 37.8% vaginal), parity: 2.2 ± 1.4, mean age: 27.1 ± 6 yr, BMI: 25.9 ± 6.8	Supine, lower limbs flexed, and feet on a bed	- Trunk flexed, scapulae off the support, and arms along the body	Not specified
Beamish 2019 (Canada) [8]	To investigate the impact of the measurement site and task on IRD, linea alba stiffness, and distortion, and to assess the relationships between the linea alba parameters	20 women (9 nulliparas and 11 paras ≥ 1 yr after vaginal delivery: 1 primipara, 10 multiparas) with and without DRA, mean age: 31 ± 8 yr, BMI: 25.3 ± 3.9	Supine, thin pillow under the head, and arms along the body	- Head lift: head gently lifted off the pillow - semi curl-up: head and shoulders lifted and inferior angles (not the spines) of the scapulae in contact with the examination table	At the end of expiration (breathing paused for image capture)
Belo 2020 (Brazil) [61]	To determine the reliability and accuracy of IRD measurement using a caliper compared with USI	54 pregnant women at 28–41wk gestation, mean age: 24.6 ± 5.6 yr, BMI: 29.3 ± 5.1	Supine, hips and knees flexed, feet on a bed, and arms by the side	- Trunk flexed and lower borders of scapulae off the bed	During expiration
Benjamin 2020 (Australia) [67]	To determine the criterion validity of the use of a caliper, tape measure, and finger width to evaluate IRD compared with USI	50 paras ≥ 6wk postpartum (26 primiparas, 24 multiparas, delivery: 21 vaginal, 19 cesarean, and 10 vaginal with instrumentation, age: 32.8 ± 4.8 yr, BMI not reported	Supine, knees flexed to 90°, arms by the side	- Modified abdominal sit-up: arms crossed over the chest, head raised, and inferior angles of the scapulae off the surface	At the end of expiration
Castellanos-López 2022 (Spain) [57]	To compare anteroposterior diameter of the levator ani hiatus, abdominal muscle thickness, and IRD between women with and without dyspareunia	32 non-pregnant, non-postpartum women: 16 with dyspareunia (mean age: 25.4 ± 3.4 yr, BMI: 21.7 ± 2.9), 16 without dyspareunia (mean age: 26.7 ± 4 yr, BMI: 22.8 ± 1.7)	Supine, pillow under the head, hips and knees flexed	- Abdominal contraction: arms crossed over the chest, upper trunk raised, spines of the scapulae off the examination table	Breathing phase considered irrelevant
Chiarello 2013 (USA) [62]	To assess the concurrent validity of the use of digital calipers and USI for the measurement of IRD	56 subjects (11 men, mean age: 37.5 ± 9.8 yr, BMI: 30 ± 3.9; 22 nulliparas, mean age: 28.5 ± 6 yr, BMI: 23.4 ± 4; 23 paras, mean age: 39.6 ± 9.8 yr, BMI: 22.6 ± 1.8	Hook lying, pillow under the head, arms by the side	- Partial curl-up: arms crossed over the chest, head and spine of the scapulae off the examination table	Not specified
Chiarello 2016 (USA) [9]	To determine whether IRD decreases with active abdominal contraction in men and nulliparous and parous women	56 subjects (11 men, mean age: 37 ± 10 yr, BMI: 30 ± 3.9; 22 nulliparas, mean age: 27.9 ± 5.9 yr, BMI: 23.4 ± 4; 23 paras, mean age: 39.2 ± 9.7 yr, BMI: 22.6 ± 1.8	Same as Chiarello (2013) [62]	Same as Chiarello (2013) [62]	Not specified
Coldron 2008 (UK) [16]	To compare the RA size and shape and IRD between day 1 and month 2, 6, and 12 postpartum, and between paras and nulliparas	72 primiparas, 43 multiparas at day 1 and month 2, 6, and 12 postpartum, mean age: 32 yr (19–46 yr, and 69 nulliparas, mean age: 27 yr (18–45 yr), BMI not reported	Crook lying, knees flexed over two pillows	Not applicable	Not specified
Corvino 2019 (Italy) [10]	To categorize the anatomical variations of DRA	92 women: 10 without DRA and 82 with DRA (5 nulliparas; 77 paras, parity: 1–4, last delivery: 48 vaginal, 29 cesarean), mean age: 35 yr (30–51 yr), BMI not reported	Supine, pillow under head, knees slightly flexed, and arms along the trunk	Not applicable	In a neutral moment immediately after expiration
Crommert 2021 (Sweden) [68]	To investigate how physical and psychological factors are linked to disability attributed to symptoms from an increased IRD in paras	139 paras at 1–8 yr since last delivery with IRD ≥ 2 finger widths (38% with at least one cesarean section), mean age: 37 ± 4.9 yr, BMI: 23.9 ± 3.5	Supine, knees flexed to 90°	Not applicable	At the end of expiration
Da Cuña-Carrera 2021 (Spain) [11]	To assess the IRD at rest and during abdominal crunch, abdominal crunch with TrA preactivation, and abdominal hypopressive exercise (AHE)	98 healthy subjects: (64 non-pregnant nulliparas and 32 men), mean age: 22.4 ± 3.6 yr, BMI: 22.8 ± 2.7	Supine, knees flexed to 90° , feet supported, and arms along the body	- Abdominal crunch: arms crossed over the chest, head and scapulae off the surface - Abdominal crunch + TrA preactivation - AHE: arms above the shoulders; after deep exhalation, breath-holding with rib expansion	At the end of expiration
Depledge 2021 (New Zealand) [64]	To determine the (immediate) effect of four abdominal exercises, Tubigrip and taping in reducing DRA at 3 wk postpartum	29 paras with DRA after vaginal delivery (mean age: 32 ± 4.6 yr, days postpartum: 21 ± 4, body height: 166 ± 5.9 cm, body mass: 65 ± 7.9 kg)	Crook lying, hips, and knees flexed to 90°	- ADIM + PFM activation - Curl-up: head, scapulae off the examination table, hands along thighs directed toward the knees - Sahrmann early level single leg raise with ADIM; supine, one leg flexed (90° hip flexion) - modified McGill side-lying plank: hip off the examination table to align trunk and thigh - ADIM, curl-up + Tubigrip, taping	Immediately after relaxed expiration (in a stable position held for 3–4 s)
Eisenberg 2021 (Israel) [27]	To investigate the clinical and morphological relationships between DRA and pelvic floor trauma in primiparas	36 primiparas (vaginal delivery), with birth-related pelvic floor trauma: 18 with DRA, mean age: 27.8 ± 4.7 yr, BMI: 23.7 ± 4.1, and 18 without DRA, mean age: 26.8 ± 4.2 yr, BMI: 23.7 ± 3.5	Supine, knees and pelvis flexed to 90°	- Abdominal curl: head and shoulders lifted, spines of the scapulae off the examination table, hands reaching the knees	At the end of expiration
Fan 2020 (Italy and Canada) [12]	To investigate differences between abdominal muscles and fasciae in women, depending on delivery mode and in comparison with nulliparas	36 women: 23 primiparas ≥ 2 yr after delivery (13 cesarean, mean age: 41.7 ± 6.1 yr, BMI: 23.7 ± 2.5 and 10 vaginal, mean age: 47 ± 15.2 yr, BMI: 21.5 ± 2.8 and 13 nulliparas, mean age: 27.1 ± 14.2 yr, BMI: 21.6 ± 1.4	Supine	Not applicable	At the end of expiration
Fernandes da Mota 2015 (Portugal and Norway) [15]	To assess the prevalence and risk factors of DRA in pregnant/postpartum women and their relationships with lumbopelvic pain	84 primiparas (at 35wk gestation, 6–8, 12–14, and 24–26wk postpartum; delivery: 52 vaginal, 32 cesarean); at 6mo postpartum: 33 with DRA, mean age: 31.6 ± 2.2 yr, BMI: 22.3 ± 3.7, and 51 without DRA, mean age: 32.5 ± 2.9 yr, BMI: 22.5 ± 3.2	Same as Mota (2012) [49]	Not applicable	At the end of expiration
Gillard 2018 (UK) [63]	To assess the effect of posture and the measurement site on IRD, and to assess the reliability of the measurement in paras	41 women (12 primiparas and 29 multiparas) between 2mo and 28 yr (mean, 9.8 ± 8.9 yr) after vaginal delivery, mean age: 43 ± 9 yr, BMI: 25.2 ± 4	Crook lying, pillow under the head, legs hip-width apart, knees flexed to 90°, feet facing forward, and mid-pelvic alignment	Postural muscle tone: - Sitting position: feet flat on the floor, arms resting on the thighs, and mid-pelvic alignment - standing position: legs hip-width apart, arms along the body, and mid-pelvic alignment	At the end of expiration
Gluppe 2020 (Norway) [13]	To investigate the immediate effects of abdominal and PFM exercises on IRD in paras with DRA	38 paras > 6wk postpartum with DRA [4 primiparas and 34 multiparas (parity: 2–4), after vaginal and cesarean deliveries], mean age: 36.2 ± 5.2 yr, BMI: 23.2 ± 3.6	Supine, hips and knees flexed, and feet on a table	Head lift, curl-up, PFM contraction, PFM contraction + curl-up, maximal ADIM, PFM contraction + maximal ADIM, pelvic tilt, and twisted curl-up (to the left and the right)	At the end of expiration
He 2021 (China) [65]	To determine the utility of shear wave elastography in assessing abdominal muscles in women with and without DRA	36 postpartum women with DRA (32 cesarean, 4 vaginal delivery), mean age: 28.6 ± 3.7 yr, pre-pregnancy BMI: 20.2 ± 2.0, parity, days postpartum not specified; 24 nulliparas, mean age: 26.8 ± 4.7 yr, BMI: 20.6 ± 2.1)	Supine, arms across the chest	Not applicable	Not specified
Hills 2018a (Canada) [53]	To determine the influence of the transducer tilt in cranial and caudal directions on IRD measurement	15 paras (5 primiparas, 10 multiparas, delivery: 9 vaginal, 6 cesarean; 7/15 with DRA), mean age: 39.4 ± 8 yr, BMI: 29.2	Supine and legs extended	- Head lift: head lifted with the neck in line with the spine, and the scapula in contact with the examination table	Not specified
Hills 2018b (Canada) [17]	To compare the trunk muscle function between women with and without DRA at 1 yr postpartum	40 primiparas at 1 yr postpartum (delivery: 31 vaginal, 9 cesarean): 18 with DRA, mean age: 31.9 ± 3.6 yr, BMI: 25.1 ± 5.6, and 22 without DRA, mean age: 31.2 ± 4.5 yr, BMI: 24.1 ± 3.9	Supine, knees and hips flexed, and arms by the side	Not applicable	At the end of tidal expiration
Iwan 2014 (New Zealand) [47]	To assess the reliability of IRD measurement using high- and low-resolution USI, and to compare measurements by novice and experienced sonographers	30 subjects: 14 men, mean age: 24.2 ± 8.3 yr, BMI: 24.4 ± 2.5, and 16 women (13 nulliparas, mean age: 21.8 ± 1.9 yr, BMI: 23.9 ± 2.8, and 3 postpartum, mean age: 36 ± 6.7 yr, BMI: 21.5 ± 2.4	Supine, pillow under head, knees flexed to 90°, feet on a table, and arms along the body	- Partial curl-up: arms crossed over the chest and head and scapulae off the examination table	At the end of expiration
Keshwani 2015a (Canada) [48]	To investigate the intrarater between-session reliability of IRD measurement	20 primiparas, multiparas with DRA at 3mo to 17 yr (mean: 3 yr) since last delivery (9 cesarean), mean age: 36 ± 6 yr, BMI: 26.1 ± 7	Supine and pillow under the head	- Head lift: with the neck in line with the spine, head gently lifted off the pillow (head touching the pillow but not supported)	Not specified
Keshwani 2015b (Canada) [54]	To investigate the criterion-related validity and reliability of IRD measurement using extended field of view (standoff pad and panoramic mode) in paras	21 non-pregnant primiparas and multiparas with IRD ≤ 3 finger widths (to visualize the entire IRD with conventional USI), mean age: 40 ± 6 yr, BMI: 22.8 ± 1.9	Supine, pillow under the head, and arms by the side	Not applicable	During expiration
Keshwani 2016 (Canada) [55]	To investigate the interrater reliability of IRD measured at rest and during head lift in paras	17 primiparas and multiparas at 6mo to 25 yr (mean: 7 yr) since last delivery; mean age: 38 ± 8 yr, BMI: 26.4 ± 7.3	Supine, pillow under the head, neck in line with the spine	Same as Keshwani (2015a) [48]	Not specified
Keshwani 2018 (Canada) [20]	To investigate the relationship between IRD and symptom severity in the early postpartum period	32 postpartum primiparas with DRA detected at 3–4wk after vaginal delivery; mean age: 32 ± 2 yr, BMI: 25 ± 3.7	Supine, pillow under the head, and arms by the side	Not applicable	Not specified
Keshwani 2019 (Canada) [33]	To explore the feasibility of abdominal binding and trunk exercises for the management of DRA	29 primiparas with DRA (first assessment at 3–4wk postpartum); mean age: 31.5 yr, BMI: 24.7	Supine	Not applicable	Not specified
Kim 2022 (Korea) [69]	To investigate the efficacy of 6-wk core stabilization exercise program conducted in person and through real-time video conferencing on IRD and abdominal muscle thickness in women with DRA at 6-12mo postpartum	37 DRA women 6-12mo postpartum [online group (9 vaginal, 10 cesarean delivery), mean age: 31.7 ± 3.9 yr, BMI: 22 ± 1.9; direct contact group (6 vaginal, 12 cesarean delivery), mean age: 32.7 ± 2.5 yr, BMI: 22.8 ± 1.9]; parity not specified	Supine	Not applicable	During normal respiration
Lee 2016 (Australia and Canada) [34]	To investigate IRD and linea alba distortion during curl-ups performed naturally and with TrA preactivation	26 DRA subjects: 25 paras and 1 nullipara; mean age: 34 ± 4 yr, BMI: 20.6, and 17 non-DRA subjects: 11 nulliparas, mean age: 25 ± 2 yr, BMI: 20.4, and 6 males, mean age: 28 ± 3 yr, BMI: 23.5	Supine, pillow under the head, hips and knees flexed, feet on a table, and arms by the side	- Curl-up: head and neck lifted, top of the scapulae off the bed, and arms along the body - Curl-up + preactivated TrA by gentle PFM contraction	Not specified
Li 2022 (China) [66]	To investigate the effects of progressive yoga exercise program on IRD among women in the early postpartum period	116 primiparas, vaginal delivery [63 yoga exercisers (mean age: 31, BMI: 21.4 ± 2); 53 controls (mean age: 30, BMI: 21.7 ± 2.1)], IRD assessed at postpartum wk 6 and 12	Supine, relaxed abdominal muscles	Not applicable	Not specified
Liaw 2011 (Taiwan) [18]	To investigate IRD natural recovery and abdominal muscle strength in women postpartum and to examine the relationship between IRD and abdominal muscle function	30 paras (17 primiparas, 13 multiparas) at 7wk (4–8 wk) and 6mo (6–8mo) after vaginal delivery, mean age: 32.1 ± 3 yr, BMI: 21.5 ± 2.8; 20 nulliparas, mean age: 31.9 ± 4.1 yr, BMI: 20.7 ± 2.7	Supine and 2 pillows under the knees	Not applicable	At the end of expiration
Mota 2012 (Portugal and Norway) [49]	To evaluate the test–retest and intrarater reliability of IRD measurement (during rest and a specific task), and to investigate IRD in postpartum women	24 women: 12 postpartum (< 6mo), mean age: 31.2 yr (26–36 yr), BMI: 24 (20.8–28.5); 12 with parity 0–2, mean age: 29.9 yr (16–55 yr), BMI: 21.5 (18.9–24.6)	Supine, knees flexed to 90°, feet on a table, and arms along the body	- Abdominal crunch: head and shoulders raised upward, scapulae off the examination table - ADIM: after inhalation, abdominal muscles drawn in toward the spine with exhalation	At the end of expiration
Mota 2013 (Portugal and Norway) [71]	To evaluate the reliability of IRD examination with abdominal palpation and compare palpation with USI	20 paras, mean parity: 0.7 (0–2), age: 29.3 yr (16–49 yr), BMI: 23 (18.9–28.5); 12/20 in the postpartum period	Same as Mota (2012) [49]	- Abdominal crunch: head and shoulders raised upward, scapulae off the examination table	At the end of expiration
Mota 2015 (Portugal and Norway) [35]	To evaluate the immediate effect of drawing-in and abdominal crunch exercises on IRD in pregnancy and postpartum	84 primiparas (at 35–41wk gestation and 6–8, 12–14, and 24–26wk postpartum; delivery: 52 vaginal and 32 cesarean), mean age: 32 yr (25–37 yr), BMI not reported	Same as Mota (2012) [49]	- Abdominal crunch: head and shoulders raised upward (while exhaling), scapulae off the table - ADIM: after inhalation and exhalation, and the navel drawn in toward the spine	Immediately at the End of expiration
Mota 2018 (Portugal and Norway) [72]	To establish “normal” IRD values in primiparas during pregnancy and postpartum	84 primiparas (at 35–41wk gestation and 6–8, 12–14, 24–26 wk postpartum; delivery: 52 vaginal and 32: cesarean), mean age: 32.1 ± 2.7 yr; BMI not reported	Same as Mota (2012) [49]	Not applicable	At the end of expiration
Morales 2018a (Spain) [43]	To compare the perimuscular connective tissue and IRD between elite and amateur basketball players	22 men (11 elite and 11 amateur basketball players), mean age: 21 ± 6 yr, BMI: 22.6 ± 2.6	Supine	Not applicable	At the end of expiration
Morales 2018b (Spain) [58]	To assess abdominal muscle thickness and IRD during the drawing-in maneuver with a proprioceptive Stabilizer	41 healthy subjects, mean age: 31.9 ± 4.5 yr, BMI: 22.9 ± 2.7	Supine	- ADIM (with the use of Stabilizer)	At the end of expiration
Nanikawa 2021 (Japan) [50]	To assess the reliability of abdominal wall perimuscular connective tissue measurement	38 healthy men, mean age: 21.6 ± 0.5 yr, BMI not reported	Supine and arms relaxed	Not applicable	At the end of expiration
Pascoal 2014 (Portugal) [36]	To determine the effect of abdominal muscle isometric contraction on IRD in postpartum women	10 primiparas (1–5mo postpartum, delivery: 9 vaginal and 1 cesarean), mean age: 30 ± 4 yr, BMI: 24.1 ± 7.0, and 10 nulliparas, mean age: 28 ± 2 yr, BMI: 21.7 ± 5.3	Supine, knees flexed to 90° (crook lying), feet on a table, and arms along the trunk	- Abdominal crunch: head and shoulders raised upward, scapulae off the examination table, and fingertips touching knees	At the end of expiration
Romero-Morales 2018 (Spain) [59]	To compare RA thickness and IRD between patients with Achilles tendinopathy (AT) who underwent an eccentric exercise (EE) program with vibration and EE program with cryotherapy	61 subjects with AT randomly divided; grade I: 4 men, 26 women receiving EE + vibration, mean age: 41.1 ± 8.2 yr, BMI: 25.2 ± 2.5, gr II: 5 men, 26 women receiving EE + cryotherapy, mean age: 42.1 ± 9.2 yr, BMI: 24.8 ± 2.4	Supine	- Calf muscle maximal isometric contraction	At the end of expiration
Romero-Morales 2019 (Spain) [44]	To compare and quantify IRD and abdominal muscle measures between subjects with and without Achilles tendinopathy (AT)	143 subjects (71 with AT, mean age: 45.1 ± 12.7 yr, BMI: 24.8 ± 2.1, and 72 without AT, mean age: 37.6 ± 11.9 yr, BMI: 23.9 ± 3.7	Supine	Not applicable	Not specified
Romero-Morales 2020 (Spain) [45]	To compare abdominal muscles thickness and IRD in women with and without primary dysmenorrhea (PD)	39 women (19 with PD, mean age: 20 ± 4 yr, BMI: 21.6 ± 3.3 and 20 without PD, mean age: 22.5 ± 7 yr, BMI: 21.5 ± 3.7	Supine, hips and knees flexed, and arms along the body	Not applicable	At the end of expiration
Sancho 2015 (Portugal and Norway) [73]	To compare IRD at rest between women who delivered vaginally and by C-section; to describe the effect of different abdominal exercises on IRD	38 postpartum primiparas after singleton gestation (23 after vaginal delivery, mean age: 31.2 ± 3.6 yr, BMI 22.9 ± 2.7, and 15 after cesarean section, mean age: 32.3 ± 4.4 yr, BMI: 22.8 ± 2.8)	Supine, knees flexed to 90°, feet on a table, and arms along the body	- Abdominal crunch: head, shoulders raised upward, scapulae off the table, fingertips touching the knees - ADIM: after inhalation, abdominal muscles drawn in toward the spine with exhalation - ADIM + abdominal crunch	Immediately at the end of expiration
Starzec-Proserpio 2022 (Poland) [70]	To evaluate the differences between early postpartum women with and without pelvic girdle pain (PGP) regarding pubic symphysis separation, IRD, and pain catastrophizing	105 women 24-72 h after vaginal delivery, 35 with PGP (mean age: 32.9 ± 4.5 yr, pre-pregnancy BMI: 24.5 ± 4.5) and 70 without PGP (mean age: 33.0 ± 4.2 yr, pre-pregnancy BMI: 22.5 ± 3.2), each group parity: 1.8 ± 0.9)	Supine	- Curl-up: head and upper torso raised, scapulae off the table	Not specified
Theodorsen 2019 (Norway) [37]	To assess the effect of the PFM and TrA muscles contraction on IRD in postpartum women with DRA	38 women 0–26wk postpartum (parity: 1–3) with DRA (delivery: 31 vaginal, 4 vaginal/forceps, 3 cesarean; 4 multiple births), mean age: 34.6 ± 4.0 yr, BMI: 24.2 ± 3.3	Supine, pillow under head, knees hip-width apart, flexed to 90°, feet on a table, and arms along the body	- PFM contraction - ADIM - PFM contraction + ADIM	At the end of expiration
Theodorsen 2022** (Norway) [75]	To investigate the effect of a specific exercise program during pregnancy on DRA after the 12-wk intervention and postpartum	Of 100 gravidas with DRA at gestational wk 24 (primigravidas and multigravidas), 50 will be assigned to exercise group and 50 to non-intervention group; IRD to be measured at 24 and 37wk gestation, and at 6wk, 6 and 12mo postpartum	Supine, head on a pillow, arms alongside the body, knees hip-width apart, bent to 90°, feet on a table	Not applicable	Immediately at the end of expiration
Whittaker 2013 (UK and Canada) [46]	To compare the resting thickness of the abdominal muscles, perimuscular connective tissue, and IRD in persons with and without lumbopelvic pain (LPP)	50 male and female subjects: 25 with LPP, mean age: 46.6 ± 8 yr, BMI: 24.0 ± 3.5, and 25 without LPP, mean age: 36.3 ± 9.4 yr, BMI: 23.5 ± 2.5	Supine	Not applicable	At the end of expiration

*Body position during an assessment at rest and/or the basic starting position for the assessment during specific muscle activity/task (except assessment in the sitting and standing positions [63])

**Study protocol (presenting research project). DRA: diastasis recti abdominis; RA: rectus abdominis; TrA: transversus abdominis; PFM: pelvic floor muscles; and ADIM: abdominal draw-in maneuver

**Table 3:** Characteristics of the examiners, ultrasonographic imaging (USI) systems, and inter-recti distance (IRD) measurement methods in 49 study protocols

	Examiner’s profession and experience	Ultrasonographic scanner and transducer	IRD measurement site	Use of cutoff values for “normal” IRD or DRA	Number of images per site	Methods of image processing and measurement
Abuín-Porras 2019 (Spain) [42]	PT with USI experience	LOGIQ S7, XDclear, GE Healthcare; Little Chalfont, UK; 10- to 13-MHz linear probe, 55 mm; B-mode	just under the navel	Not applicable	Mean IRD of 3 images	Offline measurement with ImageJ software (Research Services Branch, National Institute of Health, Bethesda, MD, USA)
Arranz-Martín 2022 (Spain/ Canada) [74]	A women’s health PT with > 5 yr of experience in abdominal and perineal USI	Mindray 7; 12-MHz 30-mm linear probe (Mindray L14-6NS, Shenzhen, China); B- mode	2 cm below and above the navel center, the midpoint between the navel center and xiphoid	Not applicable; LA distortion assessment	Mean IRD of 2 images per site and task	measurement with digital caliper
Balasch-Bernat 2021 (Spain) [56]	Two PTs with 10 yr of clinical experience in women’s health and rehabilitative USI	Samsung HS30, (Samsung Medison co., LTD, Gangwon-do, Korea); linear probe LN 5–12; B-mode; panoramic mode*	2 cm above the superior navel border and 2 cm below the inferior navel border	Not applicable	Mean IRD of 3 images per site and task	Not specified
Barbosa 2013 (Brazil) [60]	Not specified	Medison SonoAce 8000, Korea; 5- to 7-MHz linear probe	3, 6, 9, 12 cm above the navel	Not applicable	Not specified	Not specified
Beamish 2019 (Canada) [8]	PT with postgraduate training, > 400-h experience in B-mode USI of the abdominal muscles (two PT students after 10-h training and 10-h practice made offline measurements, intrarater reliability: ICC = 0.96–0.98, interrater reliability: ICC = 0.94–0.95	SuperSonic Aixplorer UltraFast (SuperSonic Imagine, Aix-en-Provence, France); 10-MHz linear probe (SL15-4); B-mode; shearwave elastography	3 and 5 cm above and at the superior navel border	DRA = IRD > 22 mm at 3 cm above the umbilicus, DRA = mean IRD > 22 mm across 3 sites; LA stiffness and distortion assessment	Mean IRD of 3 images per site and task	Video and image capturing; image processing with the SuperSonic Aixplorer software
Belo 2020 (Brazil) [61]	Examiner who was previously trained	Philips HD3xe; 5- to 9-MHz linear probe	3 cm above, 2 cm below, and at the navel	DRA = IRD > 22 mm at 3 cm above, > 16 mm at 2 cm below, and > 20 mm at the navel	mean IRD of 3 images	Not specified
Benjamin 2020 (Australia) [67]	PT after a 2-h ultrasonography training session by an experienced sonographer; 16 h of practice in USI of IRD	GE Voluson I; 3- to 8-MHz linear probe, 38.1 mm (9 L)	4.5 cm above and at the upper border of the navel, 4.5 cm below and at the lower border of the navel	DRA = IRD > 22 mm; ≥ 2 fingers width	2 images	Offline measurement
Castellanos-López 2022 (Spain) [57]	An expert in USI with several specialization courses and 5 yr of experience	LOGIC F6, GE Healthcare, Chicago, IL, USA); 6- to 13-MHz linear probe, B-mode	Same as Chiarello (2013) [62]	Not applicable	Not specified	Not specified
Chiarello 2013 (USA) [62]	Examiner with advanced training and 7 yr of clinical USI use; intrarater within-session reliability: ICC = 0.90–0.98	LOGIQ Book XP, GE Healthcare, Waukesha, WI; 5- MHz curvilinear probe; B-mode	4.5 cm above and below the navel midpoint	Not applicable	Not specified	On-screen measurement
Chiarello 2016 (USA) [9]	same as Chiarello (2013) [59]	same as Chiarello (2013) [62]	Same as Chiarello (2013) [62]	Not applicable	Not specified	Same as Chiarello (2013) [62]
Coldron 2008 (UK) [16]	Not specified	SSD, Aloka Co. Ltd., Mitaka-shi, Tokyo, Japan; 5-MHz linear probe (11-cm footprint)	Bottom transducer edge placed just cephalad to the navel	Not applicable	Mean IRD of 2 images	Offline measurement with on-screen calipers using USICA software, Dept. of Medical Physics, St. George’s Hospital, London
Corvino 2019 (Italy) [10]	Two operators with 33 and 13 yr of experience with USI, respectively. The operators had specific training in evaluating DRA	Voluson E8, GE Healthcare, and RS85 Samsung Healthcare; broadband probes, typically 10-MHz; for DRA > 4 cm: trapezoid, DRA > 5 cm: extended field of view*	3 cm above and below the navel (entire midline checked to identify the DRA pattern)	DRA = IRD > 20 mm at rest; DRA patterns: only above or below the navel, at the navel level, along the entire midline but wider above or below the navel	Mean IRD of 3 images per site	not specified
Crommert 2021 (Sweden) [68]	Not specified	LOGIQ-e R7, GE, Boston, MA; 12-MHz linear probe, 47 mm; B-mode; panoramic function*	4.5 cm above the navel center	increased IRD = IRD ≥ 2 fingers width	Mean IRD of 3 images	Offline analysis with custom-written script in MATLAB (MATLAB R2019a, MathWorks, Natick, MA, USA)
Da Cuña-Carrera 2021 (Spain) [11]	PT with knowledge of USI and experience in IRD measurement	SonoSite M-Turbo; 5- to 10-Mz linear probe; B-mode	Just above the navel (U point), halfway between the U point and xiphoid	Not applicable	Not specified	Frozen image on-screen measurement with transversal caliper
Depledge 2021 (New Zealand) [64]	PT experienced in USI, participated in a reliability study on IRD measurement with USI, ICC > 0.91 (Iwan, 2014 [44])	Philips iU22; 4- to 12-MHz linear or 4- to 9-MHz curvilinear probe (Philips Med. Syst. Co., Eindhoven, NL); B-mode	Same as Mota (2012) [49]	DRA = IRD > 2 fingers width	Mean IRD of 2 images	Not specified
Eisenberg 2021 (Israel) [27]	A physician specializing in gynecological USI	Voluson 730, GE Medical Systems, Zipf, Austria; probe not specified	Upper margin of the navel, 3 cm above, and 2 cm below the navel	DRA = IRD ≥ 22 mm at 3 cm above the navel, ≥ 20 mm at the upper margin of the navel, and/or ≥ 16 mm at 2 cm below the navel	Not specified	ARCHIVED data sets were analyzed using the proprietary software 4-D VIEW (GE Medical Systems)
Fan 2020 (Italy and Canada) [12]	PT with 5-yr experience in musculoskeletal USI	Esaote MyLab Seven (Esaote SpA, Genova, Italy); 6- to 18-MHz linear probe, 37 mm	2 cm above the navel	Not applicable	Not specified	Not specified
Q	PT with specific training in image capturing and measuring IRD	LOGIQ-e, GE; 4- to 12-MHz linear probe, 39 mm; B-mode	2 cm below the navel center	DRA = IRD > 16 mm at 2 cm below the navel center	Not specified	Images exported in DICOM format, processing as by Mota (2012)
Gillard 2018 (UK) [63]	PT, > 12mo of experience in USI, training on a national medical US society program; within- and between-session intrarater reliability: ICC = 0.90–0.99	Mindray DP50; 5-MHz linear probe, 53 mm (75L53 EA); B-mode	One-third of the xiphoid-navel distance, just superior to the navel, half of the navel-pubis distance	Not applicable	Mean IRD of 2 images per site and postural position	Offline measurement on JPEG images with bespoke MATLAB image processing software (ver. 7.1)
Gluppe 2020 (Norway) [13]	PT after specific training in USI of the pelvic floor and abdomen	LOGIQ-e R7, GE Healthcare; 5- to 13-MHz wideband linear probe, (GE > 12L-RS); panoramic mode*	2 cm above and below the navel center	DRA = IRD ≥ 2 fingers width, protrusion during curl-up; IRD ˃ 25 mm at 2 cm above/below navel	1 image per site and condition	Offline analysis with software program (MicroDicom)
He 2021 (China) [65]	A senior radiologist with 10 yr of experience in abdominal and musculoskeletal USI	Aixplorer; linear probe (SL10-2), Supersonic Imagine, FR); B-mode	Subxiphoidal, epigastric, umbilical, infraumbilical, suprapubic (International Endohernia Society, Reinpold, 2019 [24])	DRA = IRD ≥ 2 fingers width (in crook lying, arms crossed over the chest); DRA patterns: same as Corvino (2019) [10]	Not specified	Measurements with an on-screen caliper
Hills 2018a (Canada) [53]	PT with postgraduate training in musculoskeletal USI and > 50 h of experience in USI of the abdominal muscles	Voluson-i (GE Healthcare, Mississauga, Ontario, Canada); 10-MHz linear probe, 53 mm (9L-RS); B-mode; trapezoid mode*	3 and 5 cm above the navel	DRA = IRD > 20 mm	2 images per probe position and task	Offline measurement using Image J, version 1.46r (National Institutes of Health, Bethesda, MD, USA)
Hills 2018b (Canada) [17]	PT with postgraduate training in musculoskeletal USI, > 100 h of experience in USI of the abdominal muscles	Same as Hills (2018a) [53]	Superior navel border; 3 and 5 cm above the navel	DRA = IRD > 22 mm at 3 cm above navel and at least one other site; mean IRD of 3 sites > 20 mm	Mean IRD of 3 images per site	As by Hills (2018a) [53]
Iwan 2014 (New Zealand) [47]	PT, 8-yr practice in USI and 4th-yr PT student after 2- × 2-h training in USI of the abdominal anatomy and IRD measurement; intrarater reliability: within-session: ICC = 0.91–0.98 for PT, ICC = 0.89–0.98 for PT student; between-session: ICC = 0.79–0.98 for PT, ICC = − 0.51 to 0.88 for PT students	Low resolution: Chison 8300 Deluxe (Chison Medical Imaging Co. Ltd., China), 7.5-MHz linear probe; high-resolution: Phillips iU22 (Royal Philips Electronics, the Netherlands), 12.5-MHz linear probe	2 cm above and below the navel	Not applicable	2 images per condition per researcher	Measurement using the digital caliper setting on the USI unit
Keshwani 2015a (Canada) [48]	PT after 16 h of formal training on musculoskeletal USI; > 100 h of clinical USI experience; trained on IRD measurement by a USI expert; intrarater between-session reliability: ICC = 0.95–0.99	Voluson i (GE Healthcare, Waukesha, WI), 3- to 10-MHz linear probe; MyLab Five (Esaote SpA, Genoa, IT), 4- to 13-MHz linear probe; acoustic standoff pad 2 × 4 cm (ATS Lab., Inc., Bridgeport, CT)*	5 and 3 cm above and at the superior border of the navel, 3 cm below the inferior border of the navel	DRA = IRD ≥ 2 fingers width at the navel (in hook lying, neck flexed)	Mean IRD of 5 images per site and condition	Offline measurement using ImageJ (National Institutes of Health, Bethesda, MD) and software with the MyLab Five system (Esaote SpA)
Keshwani 2015b (Canada) [54]	Investigator after 16 h of formal training in USI of the abdominal muscles and > 200 h of experience in IRD evaluation with USI; 20 h of experience in extended field-of-view use; between-trial reliability for conventional, standoff pad, and panoramic techniques: ICC = 0.98–0.99	LOGIQ-e, GE Healthcare, Waukesha, WI; 4- to 13-MHz linear probe, 12.7 × 47.1 mm; B-mode; acoustic standoff pad (15 × 10 × 2 cm), ATS Lab., Bridgeport, CT; panoramic mode	Superior umbilical border	Not applicable	Mean IRD of 5 images per each method	Offline measurement using ImageJ Version 1.48, National Institutes of Health, Bethesda, MD
Keshwani 2016 (Canada) [55]	Two PTs after a 16-h course in USI. Rater 1: > 100 USI evaluations of IRD; Rater 2: 10 h of training from Rater 1; interrater reliability: ICC = 0.63–0.96	Voluson i, GE Healthcare, Chalfont St. Giles, UK; 3- to 10-MHz linear probe; B-mode; acoustic standoff pad 2 × 4 cm, ATS Lab., Bridgeport, CT*	Same as Keshwani (2015a) [48]	Not applicable	Mean IRD of 5 images per site, task, and rater (mean of at least 2 images in case of poor image quality)	Offline measurement using ImageJ, National Institutes of Health, Bethesda, MD, USA
Keshwani 2018 (Canada) [20]	Registered sonographer with specific training in the measurement approach	LOGIQ-e, GE, Buckinghamshire, UK; 5- to 13-MHz linear probe; B-mode; panoramic mode*	Same as Keshwani (2015a) [48]	DRA = IRD ≥ 2 fingers width at all sites (in crook lying, head lifted off pillow)	Mean IRD of 5 images in each site	Not specified
Keshwani 2019 (Canada) [33]	Registered sonographer with > 15 yr of experience in gynecological, obstetrical, and musculoskeletal USI; > 30 h of training on the USI protocol	LOGIQ-e, GE; 4- to 13-MHz linear probe, 12.7 × 47.1 mm; B-mode; panoramic imaging*	Same as Keshwani (2015a) [48]	DRA = IRD ≥ 2 fingers width at all sites (in crook lying, head lifted off pillow)	Mean IRD of 5 images per site	Not specified
Kim 2022 (Korea) [69]	Not specified	MySono U5, Samsung Medison, Seoul, Korea, 2010; elliptical probe, B-mode, 47–63 Hz	2.5 cm above the top of the navel	DRA = IRD ≥ 2 fingers width—self-examination, verified by USI	Not specified	Measurements made with the caliper of the ultrasound apparatus
Lee 2016 (Australia and Canada) [34]	Not specified	MyLab 25, Esaote SpA, Genoa, Italy; 12-MHz linear probe; B-mode	Just above the navel (U point), halfway between the U point and xiphoid	DRA = IRD > 22 mm at 3 cm above the navel, DRA = IRD > 15 mm inferior to xiphoid; LA distortion index: the average amount of deviation of the LA path from the shortest path between its attachments	3 images per site and condition	Images captured from videos, exported to JPEG format; analyzed using ImageJ (National Institutes of Health, Bethesda, MD, USA)
Li 2022 (China) [66]	Three physicians, each with 12 yr of clinical experience	Voluson E10 (GE Healthcare, Milwaukee, WI, USA); 5- to14-MHz linear probe (ML6-15-D)	3 cm above, below, and at the navel	Not applicable	Not specified	Not specified
Liaw 2011 (Taiwan) [18]	PT with 13 yr of experience, 5 yr of assessing abdominal muscles using USI; interimage reliability: ICC = 0.91–0.97	SSD-550, Aloka Co, Tokyo, Japan; 7.5-MHz linear probe, 38 mm; B-mode	Probe lower edge: 2.5 cm above and at the upper margin of the navel, probe upper edge: 2.5 cm below and at the lower margin of the navel	Not applicable	Mean IRD of 3 images per site	Measurement with an on-screen caliper
Mota 2012 (Portugal and Norway) [49]	PT trained in IRD evaluation with USI; discussed the USI protocol and analysis, and practiced with an experienced radiologist. Intra-image reliability: ICC > 0.90, intrarater between-day reliability: ICC = 0.50–0.90	LOGIQ-e, GE Healthcare, Waukesha, WI, USA; 4- to12-MHz linear probe, 39 mm; B-mode	The bottom edge of the probe at 2 cm above and below the navel center	Not applicable	1 image per site and condition	Offline processing using a customized program, MATLAB image processing software (MathWorks, Inc., Natick, MA); images assumed as a pixel-based coordinate system
Mota 2013 (Portugal and Norway) [71]	PT trained by an experienced radiologist	GE LOGIQ-e; 4- to 12-MHz linear probe, 39 mm; B-mode	Same as Mota (2012) [49]	Not applicable	Not specified	JPG images, processing as by Mota (2012) [49]
Mota 2015 (Portugal and Norway) [35]	PT with specific training in USI, including 3 yr of experience assessing IRD	LOGIQ-e; GE Healthcare, Waukesha, WI; 4- to 12-MHz linear probe, 39 mm (fixed frequency of 12 MHz); B-mode	The bottom edge of the probe at 2 and 5 cm above and 2 cm below the navel center	Not applicable	1 image per site and condition	DICOM images, offline analysis using a customized program (MATLAB Image Processing Toolbox) as by Mota (2012) [49]
Mota 2018 (Portugal and Norway) [72]	PT with specific training in USI, including experience in assessing IRD; discussed and practiced the USI protocol and analysis with an experienced radiologist	LOGIQ-e, GE Healthcare, Waukesha, WI, USA; 12-MHz linear probe, 39 mm; B-mode	Same as Mota (2015) [35]	Determined “normal” IRD values at 5 and 2 cm above, 2 cm below the navel: at 35–41wk gestation: up to 79, 86, and 79 mm, respectively; at 24–26wk postpartum: up to 24, 28, and 21 mm, respectively	1 image per site	DICOM images, processing as by Mota (2012) [49]
Morales 2018a (Spain) [43]	PT with 3 yr of rehabilitative USI experience	Toshiba Aplio 500 Platinum, Toshiba American Medical Systems; CA, USA; 7- to 14-MHz linear probe, 40 mm (18L7PLT-1204BT); B-mode	Just under the navel	Not applicable	Mean IRD of 3 images	Offline measurement using ImageJ software (version 2.0; US National Institutes of Health, Bethesda, MD, USA)
Morales 2018b (Spain) [58]	PT with 3 yr of USI experience	LOGIQ S7, GE Healthcare, UK; 3.1- to 10-MHz linear probe, 44 mm (9L-D); B-mode	Same as Morales (2018a) [43]	Not applicable	Same as Morales (2018a) [43]	Offline measurement as by Morales (2018a) [43]
Nanikawa 2021 (Japan) [50]	PT accustomed to USI, after sufficient practice; intrarater reliability: within-day, ICC = 0.99; between-day, ICC = 0.98	Noblus, Hitachi, Ltd., Tokyo, Japan; linear L64 probe (5- to 18-MHz); B-mode	Below the navel	Not applicable	Mean IRD of 2 images	Not specified
Pascoal 2014 (Portugal) [36]	not specified	Sonoline Prime SLC, Siemens, Erlangen, Germany; 7.5-MHz linear probe, 60 mm; B-mode	lower border of the probe: just cephalad to the navel (approximately 2 cm above the navel center)	Not applicable	Not specified	Images recorded on mini DV tape, converted to JPG; semi-automated offline analysis as by Mota (2012) [49]
Romero-Morales 2018 (Spain) [59]	Not specified	LOGIQ P7, GE Healthcare; UK; 4- to 13-MHz linear probe, 38 mm (L6-12-RS)	same as Morales (2018a) [43]	Not applicable	SAME as Morales (2018a) [43]	ImageJ software (version 2.0) used for offline analysis
Romero-Morales 2019 (Spain) [44]	PT with 3 yr of experience in USI of the musculoskeletal field	LOGIQ, GE, Healthcare, UK; 4- to 13-MHz linear probe, 38 mm	Same as Morales (2018a) [43]	Not applicable	same as Morales (2018a) [43]	ImageJ software (Bethesda, MD, USA) used for offline measurement
Romero- Morales 2020 (Spain) [45]	PT with 5 yr of USI experience	LOGIQ R S7 R3 XDclear, GE Healthcare, Milwaukee, WS, USA; 5- to 15-MHz linear probe, 44 mm (GE ML6–15); B-mode	Just above the navel (according to the photo and its legend)	Not applicable	Same as Morales (2018a) [43]	Measurement using ImageJ software (version 2.0; US National Institutes of Health, Bethesda, MD, USA)
Sancho 2015 (Portugal and Norway) [73]	PT trained in image capturing and IRD measurement	LOGIQ-e; GE Healthcare, Hatfield, UK; 4- to 12-MHz linear probe, 30 mm; B-mode	2 cm above and below the navel center	Not applicable	The best of 3 images (per condition and site)	images imported in JPG format; offline semi-automated analysis as by Mota (2012) [49]
Starzec-Proserpio 2022 (Poland) [70]	Two experienced women’s health PTs trained in the musculoskeletal USI; attended a half-day training together to promote consistency and avoid bias throughout the data collection process	Voluson P6 (GE Healthcare Syst.; Chicago, IL, USA); 4- to 12-MHz, 37-mm linear probe; palpation + caliper used to measure IRD wider than the probe	2 cm above the navel	Not applicable	Not specified	The measuring feature was used: after capturing the image, an on-screen cursor was used to mark the IRD
Theodorsen 2019 (Norway) [37]	PT after specific training in USI of the pelvic floor and abdomen	Mindray M7; 5- to 10-MHz linear probe	Lower edge of the probe at 2 cm above and below the navel center	DRA = IRD ≥ 2 finger widths at or 2 cm above/below the navel; protrusion	1 image per site and condition	Image in digital format processing; use of ultrasound’s integrated measurement tool
Theodorsen 2022** (Norway) [75]	Women’s health PT with 21 yr of clinical experience; with specific training and clinical experience in USI of the pelvic floor and abdomen	Alpinion EC8 Diamond; 8-17 MHz linear probe, B-mode	Lower edge of the probe 2 cm above and below the navel	DRA = IRD ≥ 28 mm at the navel level and/or 2 cm above and below the navel (at rest) and/or abdominal protrusion	Not specified	JPG images will be transferred to a server and the measurements will be performed using MicroDicom software
Whittaker 2013 (UK and Canada) [46]	PT with 10 yr of USI experience; within- and between-day intrarater reliability of IRD measurement: ICC = 0.99	MyLab 25, Esaote North America, Inc., Indianapolis, IN; 5.0-MHz curvilinear probe, 40 mm, resolution: 1.0 mm (lateral), 0.93 mm (axial); B-mode	Just inferior to the navel	Not applicable	Mean IRD of 3 images	Offline measurement using MATLAB Ver. 7.1 software, MathWorks, Inc., Natick, MA; in consultation with a video clip of IRD

^*^This procedure/mode was used when IRD was too large to be visualized on conventional USI. **Registered study protocol (presenting research project). PT: physiotherapist; DRA: diastasis recti abdominis; LA: linea alba; DICOM: Digital Imaging and Communications in Medicine

### Synthesis of results

To present descriptions of specific aspects of the IRD measurement procedure using USI from individual publications, three reviewers (A.O.B., D.C., and M.C.) compared charted extracts, discussed uncertain issues, resolved disagreements, and jointly made summaries of the extracts. If the studies followed the measurement procedure described in previous publications, “same as….” was stated. The citations are presented in Tables 2 and 3 in alphabetical order.

## Results

### Selection of sources of evidence

**Fig. 1:**
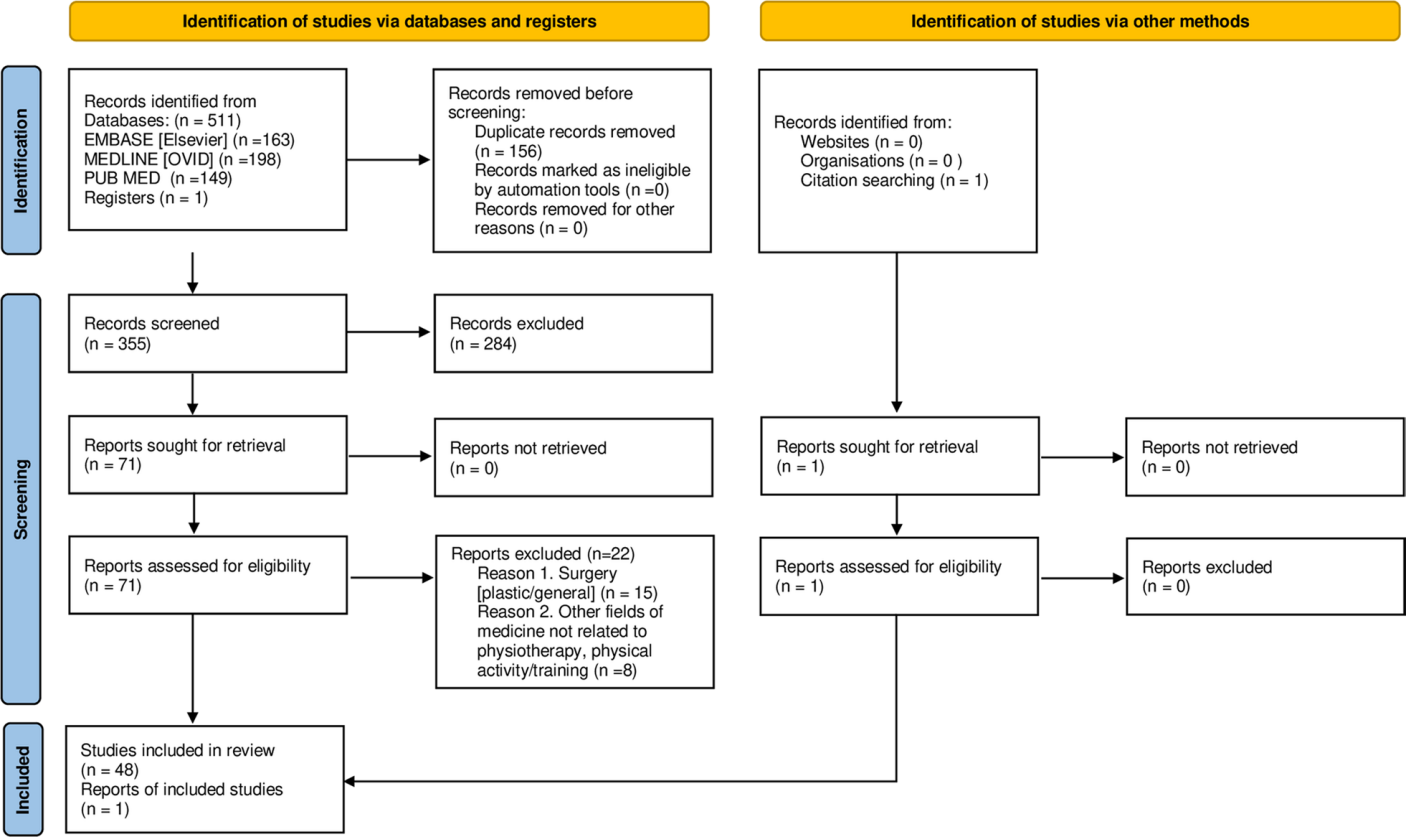
PRISMA-ScR flow diagram

Details on the selection are presented in the PRISMA flow diagram (Fig. 1).

### Characteristics of sources of evidence

All 49 included publications (published between 2008 and August 2022) were related to physiotherapy or physical exercise/training and described IRD measurements using USI.

Eight studies were from Canada [8, 17, 20, 33, 48, 53–55] and nine from Spain [11, 42–45, 56–59]. Countries with two studies were Brazil [60, 61], the USA [9, 62], the UK [16, 63], Norway [13, 37], New Zealand [47, 64], and China [65, 66]. Countries with one study were Israel [27], Australia [67], Japan [50], Italy [10], Sweden [68], Taiwan [18], Portugal [36], Korea [69], and Poland [70]. Six 2-center studies were from Portugal and Norway [15, 35, 49, 71–73], and one from Italy and Canada [12], Australia and Canada [34], the UK and Canada [46], and Spain and Canada [74]. One registered study protocol was from Norway [75].

### Results of the individual sources of evidence

Table 2 presents study objectives, populations, and IRD measurement conditions; Table 3 shows the examiners’ experience, USI systems, and IRD measurement methods.

### Synthesis of the results

The main objectives of the 49 studies are presented in Additional file 1: S6—Objectives of the Studies.

#### Population

Of the 49 publications, 36 (73.5%) concerned women in their perinatal periods: In 31, the assessment was scheduled after pregnancy/pregnancies in [8, 10, 12, 13, 16–18, 20, 27, 33, 34, 36, 37, 48, 49, 53–56, 60, 63–71, 73, 74], in four during and after pregnancy [15, 35, 72, 75], and in one during pregnancy (2.8%) [61]; 17 publications concerned women with increased IRD/DRA [8, 10, 13, 15, 17, 20, 27, 33, 34, 37, 48, 53, 64, 65, 68, 69, 75]. In 13 of the 49 studies (26.5%), the study populations comprised of men and/or women (nulliparas and/or paras): Three were validity/reliability studies [47, 50, 62], three investigated the impact of specific tasks on IRD [9, 11, 58], five assessed the IRD in medical conditions not related to pregnancy [44–46, 57, 59], and two investigated the IRD in athletes [42, 43] (Table 2).

#### Body position, muscle activity, and breathing during the IRD examination

In all 49 studies, the IRD was measured in the supine position: in 26 (53.1%), at rest and during muscle activity/task [8, 9, 11, 13, 27, 34–37, 47–49, 53, 55–60, 62, 64, 67, 70, 71, 73, 74], in 21 (42.9%), only at rest [10, 12, 15–18, 20, 33, 42–46, 50, 54, 65, 66, 68, 69, 72, 75], in one (2%), only during muscle activity/task [61], and in one (2%), in supine (at rest), and in sitting and standing [63]. One of the studies also performed measurements in side plank position [64].

Of the 27 studies that examined IRD during specific muscle activity/task in supine, some used more than one task. The tasks were head lift [8, 13, 48, 53, 55], trunk flexion with scapulae raised fully or partially off the coach (referred to as trunk flexion [60, 61], partial curl-up [9, 47, 62], semi curl-up [8, 74], curl-up [13, 27, 34, 56, 64, 70], abdominal crunch [11, 35, 36, 49, 71, 73], modified abdominal sit-up [67], and abdominal contraction [56]), transversus abdominis contraction/abdominal drawing-in maneuver (ADIM) [13, 35, 37, 49, 56, 58, 73, 74], curl-up/abdominal crunch with preactivated transversus abdominis (by ADIM [11, 73, 74] or pelvic floor muscles (PFM) contraction [34]), exclusive PFM contraction [13, 37], combined PFM contraction and curl-up [13], combined PFM contraction and ADIM [13, 37, 64], abdominal hypopressive exercise (AHE) [11, 74], pelvic tilt [13], twisted curl-up (to the left and right) [13], calf muscle maximal isometric contraction [59], Sahrmann early level single leg raise combined with ADIM, ADIM with Tubigrip and ADIM with taping, curl-up with Tubigrip, and curl-up with taping [64].

Of the 49 study protocols, 14 (28.6%) mentioned supporting the examinee’s head on a pillow during IRD measurement in the supine position [8–10, 20, 34, 37, 47, 48, 54, 55, 57, 62, 63, 75]. Thirty-one (63.3%) of the 49 protocols specified the examinees’ lower extremity position in supine: In 30, the lower extremities were flexed (for the measurements at rest [10, 15–18, 45, 63, 68, 72, 75], at rest and during specific task/tasks [9, 11, 13, 27, 34–37, 47, 49, 56, 57, 60, 62, 64, 67, 71, 73, 74], and during specific tasks only [61]), and in one they were extended (for the measurements at rest and during head lift) [53].

Of the 30 protocols using flexed lower extremities, in 26 examinees’ feet were supported on the examination table [9–11, 13, 15, 17, 27, 34–37, 45, 47, 49, 60–64, 67, 68, 71–75] (16 specified that knee flexion angle was 90° [11, 15, 27, 35–37, 47, 49, 63, 64, 67, 68, 71–73, 75]), in 3, one [56] or two pillows [16, 18] were placed under the examinees’ knees, and in one the method of obtaining lower extremity flexion was not specified [57] (Table 2).

Of the 49 studies, in 32 (65.3%), IRD images were captured during a specific moment of the breathing phase: in 27 at the end of normal expiration [8, 11–13, 15, 17, 18, 27, 35–37, 42, 43, 45–47, 49, 50, 59, 63, 67, 68, 71–73, 75], in two in a neutral moment immediately after expiration [10, 64], in two during expiration [54, 61], and in one the breathing pattern/phase was related to a specific exercise [74] (Table 2).

#### Examiners and examination tools

Of the 49 protocols, 36 (73.5%) specified the examiner’s profession: In 31 protocols, it was a physiotherapist [1, 8, 11, 12, 15, 17, 18, 35, 37, 42–50, 53, 55, 56, 58, 63, 64, 67, 70–75], in two registered sonographers [20, 33], in one physicians [66], in one a physician specializing in gynecological USI [27], and in one a radiologist [65]. Of the 49 protocols, 41 (83.7%) included information on the examiner’s training and/or experience in USI [8–13, 15, 17, 18, 20, 27, 33, 35, 37, 42–50, 53–58, 61–65, 67, 70–75] (34 described type of examiner’s training/expertise [8–13, 15, 17, 18, 20, 27, 33, 35, 37, 43, 44, 47–49, 53–56, 58, 62, 63, 65, 67, 70–75] and 25 training and/or experience duration [8–10, 12, 17, 18, 33, 35, 43–48, 53–56, 58, 62, 65, 67, 70, 74, 75]). Twelve studies reported the reliability of the IRD measurements using USI [8, 18, 46–50, 54, 55, 62–64]. (Six of them were reliability studies [47–50, 55, 63].)

Forty-eight (97.9%) of 49 protocols described ultrasound transducer type: 43 chose linear probe [8, 10–13, 15–18, 20, 33–37, 42–45, 47–50, 53–61, 63, 65–68, 70–75], three curvilinear [9, 46, 62], one both types [64], and one provided unclear information [69]. Forty-six protocols specified probe frequency (3–18 MHz); 27 provided probe size (3–11 cm) [12, 15–18, 33, 35, 36, 42–46, 49, 53, 54, 58, 59, 63, 66–68, 70–74]. Eleven studies used additional features, settings, or products (panoramic imaging [13, 20, 33, 54, 56, 68], trapezoid mode [10, 17, 53], and acoustic standoff pad [48, 54, 55]) that enabled field of view extension, one compared IRD measurements using low and high ultrasound resolutions [47] (Table 3).

#### Measurement sites

Of the 49 protocols, 16 (32.6%) used one measurement site [12, 15, 16, 36, 42–46, 50, 54, 58, 59, 68–70]; another 16 (32.6%) two sites [9–11, 13, 34, 37, 47, 49, 53, 56, 57, 62, 64, 71, 73, 75]; nine (18.4%) three sites [8, 17, 27, 35, 61, 63, 66, 72, 74]; seven (14.3%) four sites [18, 20, 33, 48, 55, 60, 67]; and one (2%) five sites [65]. However, of the 39 studies on pregnancy-related IRD, only seven (17.9%) used one measurement site [12, 15, 16, 36, 54, 68, 69], while of the 10 studies on non-pregnancy-related IRD, one measurement site was used in nine (90%) protocols [42–46, 50, 58, 59, 70].

In 26 (53.1%) of the 49 study protocols, the IRD measurements were made at the superior umbilical border (16/26, 61.5%) [8, 11, 16–18, 20, 27, 33, 34, 36, 45, 48, 54, 55, 63, 67] and/or at the inferior umbilical border (9/26, 34.6%) [18, 42–44, 46, 50, 58, 59, 67] (two studies used both sites [18, 67]), and directly at the umbilicus (3/26, 11.5%) [61, 65, 66].

In 38 (77.5%) of the 49 study protocols, more distant measurement sites were chosen: supraumbilical in 37 (97.4%) [8–13, 17, 18, 20, 27, 33–35, 37, 47–49, 53, 55–57, 60–75] and/or infraumbilical in 27 (71%) [9, 10, 13, 15, 18, 20, 27, 33, 35, 37, 47–49, 55–57, 61–65, 67, 71–75]. (26 of these protocols used both locations [9, 10, 13, 18, 20, 27, 33, 35, 37, 47–49, 55–57, 61–65, 67, 71–75].) Supraumbilical measurements were made at 2 cm [12, 13, 35, 37, 47, 49, 56, 64, 70–75], 2.5 cm [18, 69], 3 cm [8, 10, 17, 20, 27, 33, 48, 53, 55, 60, 61, 66], 4.5 cm [9, 57, 62, 67, 68], 5 cm [8, 17, 20, 33, 35, 48, 53, 55, 72], and 6, 9, and 12 cm [60] above the umbilicus; a third of the distance [63] and halfway [11, 34, 74] between the umbilicus and xiphoid, and at subxiphoid and epigastric sites [65].

Infraumbilical measurements were taken at 2 cm [13, 15, 27, 35, 37, 47, 49, 56, 61, 64, 71–75], 2.5 cm [18], 3 cm [10, 20, 33, 48, 55, 66], and 4.5 cm [9, 57, 62, 67] below the umbilicus, halfway between the umbilicus and pubis [63], and at infraumbilical and suprapubic sites [65].

Of the 38 protocols describing supra- and infraumbilical measurement sites, 37 indicated starting points to identify them: 25 precisely indicated these points [8, 9, 11, 13, 15, 18, 20, 33–35, 37, 48, 49, 55–57, 62, 64, 67–69, 71–74] (14 indicated umbilical midpoint [9, 13, 15, 35, 37, 49, 57, 62, 64, 68, 71–74], and 11 superior and/or inferior umbilical borders [8, 11, 18, 20, 33, 34, 48, 55, 56, 67, 69]); 12 less precisely indicated that the sites were measured from the umbilicus [10, 12, 17, 27, 47, 53, 60, 61, 63, 66, 70, 75] (Table 3).

#### Screening subjects for DRA

Of the 49 study designs, 19 required screening subjects for DRA/increased IRD: 12 studies chose USI for screening [8, 10, 13, 15, 17, 27, 34, 53, 61, 67, 69, 75]; however, only nine of them referred to normative values (eight [8, 15, 17, 27, 34, 53, 61, 67] referred to “normal” linea alba width values for nulliparas [76] and one [75], to “normal” IRD for postpartum primiparas [72]); nine studies used a palpatory clinical test [77, 78] for screening [13, 20, 33, 37, 48, 64, 65, 68, 69]. (Two performed screening using both USI and palpation [13, 69].) In one study, tape measurement DRA classification [79] was used for screening [13], and in three study protocols, abdominal wall protrusion [80] was an additional inclusion criterion for DRA [13, 37, 75]. Studies used different measurement conditions (i.e., body position, muscle activity, measurement sites) for screening and differently interpreted the palpatory examination outcomes. One study determined “normal” IRD values for primiparas in pregnancy and postpartum [72]. Another study proposed DRA pattern classification [10] (Table 3).

*Number of images per measurement site.* In 25 (51%) of the 49 studies, more than one image was captured at each measurement site to calculate the mean values of IRD: The mean IRD of three, five, and two images was used in 15 [8, 10, 17, 18, 42–46, 56, 58, 59, 61, 68, 73], five [20, 33, 48, 54, 55], and five [16, 50, 63, 64, 74] studies, respectively. One study used the best of three images at each measurement site [73]. Three studies just mentioned that two [53, 67] or three [34] images were taken per measurement site; five used single images [13, 35, 37, 49, 72]; and 15 did not specify the number of images per sites [9, 11, 12, 15, 27, 36, 57, 60, 62, 65, 66, 69–71, 75] (Table 3).

#### Image processing and measurement methods

In 38 (77.5%) of the 49 studies, the methods of frozen image processing and IRD measurement were described: 27 indicated offline processing (26 specified software type [13, 15–17, 27, 34–36, 42–46, 48, 49, 53–55, 58, 59, 63, 68, 71–73, 75]), three indicated the measurement tool/software integrated into the ultrasonographic scanner [8, 37, 47], and eight on-screen measurement [9, 11, 18, 62, 65, 69, 70, 74]. In four studies, the procedure included video capturing [8, 34, 36, 46] (Table 3).

## Discussion

### Summary of evidence

This scoping review aimed to present IRD measurement procedures using USI that have been used in physiotherapy-related research. Using the IRD measurement method descriptions from the 49 systematically mapped original peer-reviewed publications, the data were synthesized. The review indicates that the studies used different approaches regarding specific aspects of the IRD measurement procedure which, to a great extent, restrict or prevent cross-study comparisons. Based on the synthesis of the results, practical conclusions and recommendations on the standardization of the procedure for future physiotherapy research and clinical use have been made.

The first synthesized aspects of the IRD measurement procedure were the examinee’s body position, specific muscle activity/task, and breathing phase during image capturing. In all 49 publications, the supine position was the basic body position for IRD examination. Depending on the study aim, this position was used to measure the IRD at rest, and/or as a starting position to perform a specific task for IRD measurement during muscle activity. Several protocols included information that in the supine position, the examinee’s head was rested on a pillow. Slight head elevation improves the examinee’s comfort and should not interfere with IRD measurements. However, pillows of unspecified sizes do not allow a uniform angle of neck flexion. To standardize the head position, an examination table with adjustable headrest that can be elevated to the desired angle (i.e., 15°) might be considered.

The majority of protocols specified the examinees’ lower extremity position during the IRD examination, and in almost all of them, it was hip and knee flexion. For the examinations at rest and/or during specific tasks, lower extremity flexion was attained by supporting feet on the examination table. The knee flexion angle of 90° was the most frequent. However, in a few studies lower extremity flexion was obtained by placing one or two pillows under the examinees’ knees. To ensure reproducibility, the lower extremity position during the IRD measurement in the supine position should be described in detail. The pillow of unspecified size placed under the examinees’ knees does not allow for reproducing the lower extremity position. Instead of pillows, the examiners may consider a foam roller. For instance, a foam roller of 15 cm in diameter ensures slight hip and knee flexion and enhances the examinee’s comfort in the supine position. The position involving slight lower extremity flexion may be useful, especially if the IRD is measured only at rest.

If the IRD is measured in supine with greater hip and knee flexion angles, this position may be accompanied by reduced anterior pelvic inclination/tilt angle [81]. It should be accounted for that as a result of the increased posterior pelvic tilt, the linea alba insertion at the pubic symphysis and its origin at the xiphoid come closer, and the anterior abdominal wall structures relax. Theoretically, this position may alter the linea alba length, tension, and width; however, to the authors’ knowledge, this has not been investigated. Considering pelvifemoral motion during hip flexion [81], we recommend that researchers provide at least the magnitude of the examinee’s knee flexion angle when using the crook-lying position with feet supported on the examination table. In one study, the authors attempted to standardize the pelvic inclination and examined the IRD after teaching the examinee to maintain a neutral pelvic position [63]. The study was also the only one that used sitting and standing positions in addition to supine for IRD measurement [63]. The measurement in vertical positions is an interesting approach from the perspective of physiotherapy. It may allow to, for example, determine the effects of specific treatments in DRA subjects on their linea alba parameters during postural abdominal muscle tone and increased load on the anterior abdominal wall structures compared to relaxed supine position.

Most protocols included information that IRD images were captured during a specific moment in the breathing phase and most commonly, it was at the end of normal expiration. During resting ventilation, the abdominal cavity volume and intra-abdominal pressure are modulated depending on the respiratory phase; therefore, the tension of the anterior abdominal wall soft tissues, including linea alba, fluctuates. It has been also suggested that normal expiration is accompanied by some engagement and increased thickness of the abdominal muscles [82, 83]. As the linea alba is formed by interlacing abdominal muscle aponeuroses [6], it can be assumed that its tension and width may also change during the respiratory cycle. Ultrasound images should therefore be captured at the same phase of the breathing cycle (specified in the description of the measurement procedure). The approach to capture images at the end of normal expiration is consistent with previous recommendations on the evaluation of the abdominal muscle parameters and IRD with USI [84, 85].

In this review, the characteristics of the examination tools, selection of IRD measurement sites, and methods of DRA screening were also synthesized. Most studies used linear transducers (a few curvilinear) with frequencies ranging from 3 to 18 MHz. In over half of the protocols, transducer’s size was provided, ranging from 3 to 11 cm (most often, 4 cm). Some protocols specified additional features, settings, or products used, such as panoramic imaging, trapezoid mode, and acoustic standoff pad to extend the field of view to measure wider DRA. Acoustic standoff pad and panoramic mode have been suggested to be valid methods to measure the IRD of up to three finger widths. However, further research is needed on the validity and reliability of the extended field-of-view methods for greater IRD [54]. Sufficient training and experience in the use of panoramic USI are also of importance [86].

This scoping review indicates researchers used 1 to 5 measurement sites during their IRD assessments. Most studies that investigated pregnancy-related IRD/DRA used more than one measurement site, while the great majority of studies on individuals with other medical conditions and athletes performed measurements only at one site. As linea alba width changes along its course [6, 10, 72, 76, 87, 88], examining more than one location is important. This review confirms its relevance in research on pregnancy-related IRD/DRA.

Concomitantly, the comparison of findings across studies is only possible if IRD measurements are made at the same anatomical location. This scoping review indicates there is no consistency between study protocols regarding this aspect. The IRD was examined at the superior and/or inferior umbilical borders and at various levels (between 2 and 12 cm) above and (between 2 and 4.5 cm) below the umbilicus. A few protocols presented a valuable approach to supra- and infraumbilical site selection by taking into account the inter-individual variability in the linea alba length (i.e., the distances from the umbilicus to the linea alba origin and insertion). The measurements were performed at a third of the distance [63] or halfway [11, 34, 74] between the umbilicus and xiphoid, and halfway between the umbilicus and pubis [63].

This review also indicates the IRD was more frequently examined at the superior than at the inferior umbilical border, and above than below the umbilicus. In addition, most infraumbilical measurement sites were relatively close to the umbilicus. When choosing the measurement sites along the linea alba, the current knowledge on the measurement reliability at specific locations should be considered [47–50, 55, 63]. According to a systematic review and meta-analytical reliability generalization [7] and subsequent reliability studies [48, 63], the reliability of IRD measurements using USI is high; however, it is slightly lower for infraumbilical than for supraumbilical measurements. Infraumbilical measurements are more challenging because, at this anatomical location, the posterior rectus sheath is hardly visible [48, 89]. In women with DRA and history of cesarean section, the infraumbilical measurements using USI have been reported inaccurate compared to intraoperative surgical compass measurements. The researchers suggested that their difficulty in measuring the IRD at that location was due to the cesarean section-related fibrosis and loss of definition of the posterior layer of the recti muscles [89]. This is consistent with the cadaver study that indicated the posterior rectus sheath below the umbilicus consisted of much thinner collagen fibers than the fibers of the posterior rectus sheath above the umbilicus [6]. It is therefore possible that less frequent use of infraumbilical measurement sites indicated by this scoping review was due to the greater difficulty to measure the IRD at this location. Considering this, it is important to determine measurement reliability at specific sites before the primary investigation, as performed in several studies [8, 18, 46, 54, 62]. It should be noted that measurement reliability may also depend on the examiner’s experience [7, 47].

Most protocols that included IRD measurements away from the umbilicus specified the starting points from which the anatomical locations were measured. These starting points were the umbilical midpoint, and the superior and/or inferior umbilical borders (for locations above and/or below the umbilicus, respectively). To enable cross-study comparisons, we propose the superior umbilical border as the standard starting point to determine supra- and infraumbilical measurement sites. This would reduce possible discrepancies in determining these locations caused by inter-individual variability in the size of the umbilical ring.

**Fig. 2:**
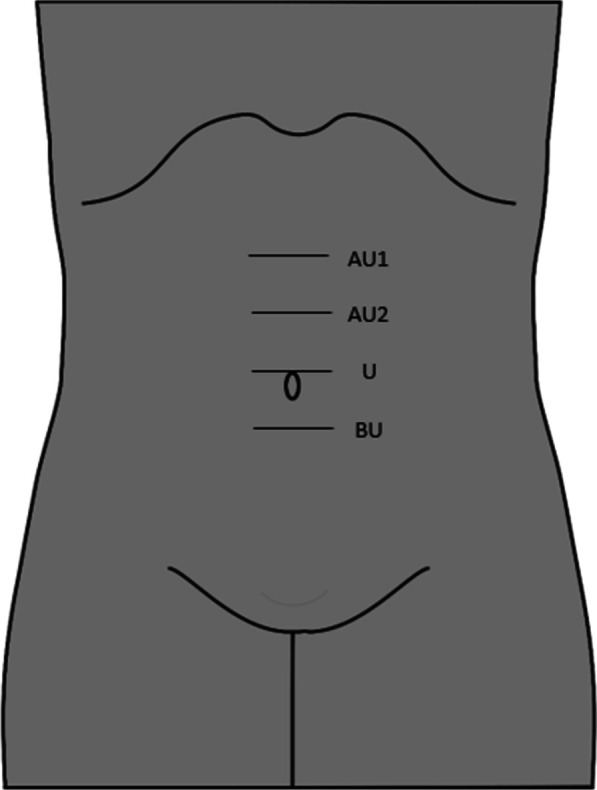
Proposed sites for inter-recti distance measurement using ultrasonographic imaging based on the scoping review results synthesis. AU1: ½ of the superior umbilical border-xiphoid distance; AU2: ¼ of the superior umbilical border-xiphoid distance; U: the superior umbilical border; and BU: ¼ of the superior umbilical border-pubis distance

Considering the need to standardize the measurement protocol to facilitate cross-study comparisons [7, 39, 40] and the synthesis of results in this scoping review, we propose four basic measurement sites:

- half of the superior umbilical border-xiphoid distance;
- a quarter of the superior umbilical border-xiphoid distance (most proximal to the umbilicus);
- the superior umbilical border;
- a quarter of the superior umbilical border-pubis distance (most proximal to the umbilicus; Fig. 2).

We recommend the locations of a quarter of the superior umbilical border-xiphoid/pubis distances as they roughly correspond to locations 4.5/5 cm above/below the umbilicus, which have been frequently applied. When choosing measurement sites in women with pregnancy-related DRA, it should be remembered that supraumbilical DRA patterns are the most common, and in the case of complete opening, the diastasis is usually wider above the umbilicus [10].

More than half of the protocols included information that 2–5 images were taken per site to calculate measurement means (most often the mean IRD of three images). In one study, the best of three images per site was used for the measurement [73]. Higher intrarater and interrater reliability and lower intrarater measurement error were demonstrated when using means of 2 or 3 consecutive measurements in determining specific abdominal muscle parameters with musculoskeletal USI [1, 90]. Therefore, to enhance measurement precision, we propose to use the mean value of three measurements taken at each anatomical location. This may be of particular importance when the assessment is performed by a less experienced examiner.

Our synthesis revealed researchers used different selection methods and criteria to identify subjects with or without DRA. Most researchers used ultrasound screening with cutoff values for “normal” linea alba width that had been determined by Beer et al. [76]. In that study, the examinees were in the supine resting position with the lower extremities extended; the measurement sites were at the xiphoid, 3 cm above and 2 cm below the umbilicus [76]. For consistency, these measurement conditions and sites should be followed during screening for DRA. However, some of the studies modified the procedure.

It is still unclear what IRD values (obtained by USI) should be considered physiological and pathological from the perspective of proper functioning of the abdominal wall structures [14, 38–40]. Normative IRD values of up to 15 mm at the xiphoid, up to 22 mm at 3 cm above the umbilicus, and up to 16 mm at 2 cm below the umbilicus were designated using the 90^th^ percentile. The investigators suggested that DRA could be anticipated beyond these values with an error rate of 10%. The study also presented the population’s extreme linea alba widths, which were 31 mm at the xiphoid, 35 mm at 3 cm above the umbilicus, and 31 mm at 2 cm below the umbilicus. They concluded that the DRA could be anticipated beyond these values without doubt [76]. Another study proposed the cutoff values for “normal” IRD in primiparas using the 80^th^ percentile of the values obtained in 84 females in their perinatal period. At six months postpartum, these values were 24 mm at 5 cm above the umbilicus, 28 mm at 2 cm above the umbilicus, and 21 mm at 2 cm below the umbilicus. The authors suggested that, in primiparas, the IRD might be considered “normal” up to values wider than for nulliparas [72]. Further investigation is warranted to clarify the DRA diagnostic criteria based on USI. Also, systematic mapping and verification of the quality of the existing studies that determined “normal” IRD cutoff values using different methods may provide additional information on what is already known and in what direction future research should proceed. Regarding physiotherapy research and practice, we suggest establishing normative values for measurement sites specified in Fig. 2. It is worth mentioning that musculoskeletal USI can also be used to inspect the entire linea alba from its origin to insertion to determine the DRA pattern and identify the location of the greatest IRD [10].

Several reviewed studies used a palpatory clinical test to screen subjects for DRA. This test is performed during abdominal muscle activation to facilitate palpation. The examinee lies in a supine position with the knees bent (at 90°) and feet resting on the examination table. The examinee is asked to raise the head and shoulders upward (until the spines of the scapulae leave the examination table). To palpate the IRD, the examiner places fingers horizontally across the midline of the examinee’s abdomen at the umbilicus. The same procedure is then repeated above and below the umbilicus [67, 71, 77, 78]. This clinical test has shown good [71] and very good [67] intrarater reliability, and moderate [71] or moderate-to-good [67] interrater reliability. The test result is considered negative when a maximum of two-finger widths can be placed between the medial parts of the right and left recti muscle bellies and/or when the fingers do not sink into the gap between the muscles, and/or no linea alba bulging or slumping is present [77–79]. This review revealed that some modifications to the test execution have been introduced by researchers, e.g., the IRD palpation during head lift instead of head and shoulder lift [20, 33, 48]. Such an approach may influence test results because abdominal muscle engagement is different between the two tasks [13, 77, 78]. Another modification concerns the interpretation of the test outcome; some studies classified two- and not more than two-finger widths as DRA [13, 20, 33, 37, 48].

Although the palpatory clinical test [77, 78] is the easiest and fastest screening procedure for DRA, the limits of agreement between this assessment method and USI are wide [67]. We believe palpatory test results of DRA = IRD > two-finger width [77, 78] should be used as indicative only. The two-finger width equals approximately 3 cm; however, it may differ especially between male and female examiners. The original interpretation of the test result as positive or negative also fails to consider the fact that normal linea alba is narrower above and below the umbilicus compared to its width at the umbilicus [6]. Therefore, IRD palpation should be combined with caliper measurement, which is a reliable and valid method [62, 67]. It would be necessary though to determine “normal” IRD values during abdominal muscle activity under standardized conditions using a caliper. Notwithstanding, the strength of the palpatory method is that it enables identifying linea alba slumping or bulging during abdominal muscle activation [79]. It also enables examination of the entire linea alba for the DRA pattern.

### Limitations

The limitation of this scoping review is that the publications were searched for in three major databases. Therefore, some studies might have been omitted. Also, only research papers published in English have been included.

## Conclusions

This scoping review indicates that most studies provided a detailed description of IRD measurement using USI. IRD examination was typically made with the subject in the supine position. A majority of the examinees had their hips and knees flexed. Most studies captured images at the end of normal expiration. Different measurement sites and starting points to determine supra- and infraumbilical sites were used. Most studies used linear transducers of widths and frequencies 3–11 cm and 3–18 MHz, respectively. To enhance the IRD measurement precision, most studies captured more than one image at each measurement site, and calculated the mean values of the measurements. Different approaches were used to screen subjects for DRA. Further investigation on USI-based measurements (following the protocol proposed in this review) is warranted to clarify the DRA diagnostic criteria. Determining “normal” IRD values using a caliper during abdominal muscle activity (following protocol for palpatory clinical test [67, 71, 77, 78]) should be also considered.

To facilitate the IRD measurement procedure reproducibility and cross-study comparisons, the following protocol standardization for the examination in supine is proposed based on result synthesis:

- the headrest of the examination table elevated by 15°; a foam roller (15 cm in diameter) under the knees or the knees flexed to 90° with the feet on the examination table;
- image acquisition at the end of normal expiration;
- measurement sites: (1) half of the superior umbilical border-xiphoid distance, (2) a quarter of the superior umbilical border-xiphoid distance (most proximal to the umbilicus), (3) the superior umbilical border, and (4) a quarter of the superior umbilical border-pubis distance (most proximal to the umbilicus; Fig. 2);
- use of mean IRD value of three images per measurement site as measurement outcome.

## Supplementary Information

**Additional file 1.** Supplementary documents: PRISMA-ScR Checklist, Publication Relevance Screening Form I, Publication Relevance Screening Form II, Explanation and Elaboration Document, Data Charting Form, and Objectives of the Studies.

## Data Availability

The data sets supporting the conclusions of this article are included within the article (Fig. 1, Tables 2 and 3).

## Abbreviations

ADIM: Abdominal draw-in maneuver
AHE: Abdominal hypopressive exercise
BMI: Body mass index
DICOM: Digital Imaging and Communications in Medicine
DRA: Diastasis recti abdominis
IRD: Inter-recti distance
LA: Linea alba
PFM: Pelvic floor muscles
PRISMA-ScR: Preferred Reporting Items for Systematic Reviews and Meta-Analyses Extension for Scoping Reviews
PT: Physiotherapist
RA: Rectus abdominis
TrA: Transversus abdominis
USI: Ultrasound imaging
