# Supplementary material for: Technical aspects of inter-recti distance measurement with ultrasonographic imaging for physiotherapy purposes: the scoping review
Source: Insights Imaging. 2023 May 18;14:92. doi: 10.1186/s13244-023-01443-4 (PMC10195962; doi:10.1186/s13244-023-01443-4)
Supplement: Supplementary file 1 — Additional file 1. Supplementary documents: PRISMA-ScR Checklist, Publication Relevance Screening Form I, Publication Relevance Screening Form II, Explanation and Elaboration Document, Data Charting Form, and Objectives of the Studies. [file 13244_2023_1443_MOESM1_ESM.pdf]

Technical aspects of inter-recti distance measurement with ultrasonographic imaging for physiotherapy purposes: The scoping review

Supplementary Material - Additional files:

S1. PRISMA-ScR Checklist

S2. Publication Relevance Screening Form I

S3. Publication Relevance Screening Form II

S4. Explanation and Elaboration Doc.

S5. Data Charting Form

S6. Objectives of the Studies

Technical aspects of inter-recti distance measurement with ultrasonographic imaging for physiotherapy purposes: The scoping review

Supplementary Material S1.

PRISMA-ScR Checklist

Preferred Reporting Items for Systematic reviews and Meta-Analyses extension for Scoping Reviews (PRISMA-ScR) Checklist

SECTION	ITEM	PRISMA-ScR CHECKLIST ITEM	REPORTED ON PAGE #
**TITLE**			
Title	1	Identify the report as a scoping review.	1
**ABSTRACT**			
Structured summary	2	Provide a structured summary that includes (as applicable): background, objectives, eligibility criteria, sources of evidence, charting methods, results, and conclusions that relate to the review questions and objectives.	1,2
**INTRODUCTION**			
Rationale	3	Describe the rationale for the review in the context of what is already known. Explain why the review questions/objectives lend themselves to a scoping review approach.	60-75
Objectives	4	Provide an explicit statement of the questions and objectives being addressed with reference to their key elements (e.g., population or participants, concepts, and context) or other relevant key elements used to conceptualize the review questions and/or objectives.	76-81
**METHODS**			
Protocol and registration	5	Indicate whether a review protocol exists; state if and where it can be accessed (e.g., a Web address); and if available, provide registration information, including the registration number.	87-90
Eligibility criteria	6	Specify characteristics of the sources of evidence used as eligibility criteria (e.g., years considered, language, and publication status), and provide a rationale.	91-97, Table 1
Information sources*	7	Describe all information sources in the search (e.g., databases with dates of coverage and contact with authors to identify additional sources), as well as the date the most recent search was executed.	99-104
Search	8	Present the full electronic search strategy for at least 1 database, including any limits used, such that it could be repeated.	105-113
Selection of sources of evidence†	9	State the process for selecting sources of evidence (i.e., screening and eligibility) included in the scoping review.	118-138
Data charting process‡	10	Describe the methods of charting data from the included sources of evidence (e.g., calibrated forms or forms that have been tested by the team before their use, and whether data charting was done independently or in duplicate) and any processes for obtaining and confirming data from investigators.	139-145
Data items	11	List and define all variables for which data were sought and any assumptions and simplifications made.	146-158
Critical appraisal of individual sources of evidence§	12	If done, provide a rationale for conducting a critical appraisal of included sources of evidence; describe the methods used and how this information was used in any data synthesis (if appropriate).	not applicable

SECTION	ITEM	PRISMA-ScR CHECKLIST ITEM	REPORTED ON PAGE #
Synthesis of results	13	Describe the methods of handling and summarizing the data that were charted.	159-164
**RESULTS**			
Selection of sources of evidence	14	Give numbers of sources of evidence screened, assessed for eligibility, and included in the review, with reasons for exclusions at each stage, ideally using a flow diagram.	Figure 1
Characteristics of sources of evidence	15	For each source of evidence, present characteristics for which data were charted and provide the citations.	170-179
Critical appraisal within sources of evidence	16	If done, present data on critical appraisal of included sources of evidence (see item 12).	not applicable
Results of individual sources of evidence	17	For each included source of evidence, present the relevant data that were charted that relate to the review questions and objectives.	Tables 2 and 3
Synthesis of results	18	Summarize and/or present the charting results as they relate to the review questions and objectives.	184-293
**DISCUSSION**			
Summary of evidence	19	Summarize the main results (including an overview of concepts, themes, and types of evidence available), link to the review questions and objectives, and consider the relevance to key groups.	294-481
Limitations	20	Discuss the limitations of the scoping review process.	482-485
Conclusions	21	Provide a general interpretation of the results with respect to the review questions and objectives, as well as potential implications and/or next steps.	486-510
**FUNDING**			
Funding	22	Describe sources of funding for the included sources of evidence, as well as sources of funding for the scoping review. Describe the role of the funders of the scoping review.	none

JBI = Joanna Briggs Institute; PRISMA-ScR = Preferred Reporting Items for Systematic reviews and Meta-Analyses extension for Scoping Reviews.
* Where *sources of evidence* (see second footnote) are compiled from, such as bibliographic databases, social media platforms, and Web sites.
† A more inclusive/heterogeneous term used to account for the different types of evidence or data sources (e.g., quantitative and/or qualitative research, expert opinion, and policy documents) that may be eligible in a scoping review as opposed to only studies. This is not to be confused with *information sources* (see first footnote).
‡ The frameworks by Arksey and O'Malley (6) and Levac and colleagues (7) and the JBI guidance (4, 5) refer to the process of data extraction in a scoping review as data charting.
§ The process of systematically examining research evidence to assess its validity, results, and relevance before using it to inform a decision. This term is used for items 12 and 19 instead of "risk of bias" (which is more applicable to systematic reviews of interventions) to include and acknowledge the various sources of evidence that may be used in a scoping review (e.g., quantitative and/or qualitative research, expert opinion, and policy document).

From: Tricco AC, Lillie E, Zarin W, O'Brien KK, Colquhoun H, Levac D, et al. PRISMA Extension for Scoping Reviews (PRISMAScR): Checklist and Explanation. Ann Intern Med. 2018;169:467–473. doi: 10.7326/M18-0850.

ELECTRONIC SUPPLEMENTARY MATERIAL

Technical aspects of inter-recti distance measurement with ultrasonographic imaging for physiotherapy purposes: The scoping review

Supplementary Material S2.

Publication Relevance Screening Form I

Publication relevance screening form: Title and abstract level*

*if you are not able to answer the questions based on title and abstract screening, please obtain the full text of the publication to make your final decision

* Wymagane

* Ten formularz zarejestruje Twoje imię i nazwisko, dlatego wypełnij swoje imię i nazwisko.

1. Please, enter the publication title *

2. Does the publication concern inter-recti distance (IRD)/linea alba width/diastasis recti abdominis (DRA) measurement with ultrasound imaging (USI)?

   - Yes
   - No

3. If your answer was "No" to question nr 2, please specify what the publication concerns, and then go to the last question nr 8

- ◯ USI not related to IRD/linea alba width/DRA measurement
- ◯ other procedures not related to IRD/linea alba width/DRA measurement
- ◯ IRD/linea alba width/DRA measurement with manual caliper
- ◯ IRD/linea alba width/DRA measurement with tape measure
- ◯ IRD/linea alba width/DRA measurement with ruler
- ◯ IRD/linea alba width/DRA measurement with magnetic resonance
- ◯ IRD/linea alba width/DRA measurement with computer tomography
- ◯ IRD/linea alba width/DRA assessment by palpation
- ◯ IRD/linea alba width/DRA intraoperative measurement

4. If your answer was "Yes" to question nr 2, does the study population concern the human adults?

- ◯ Yes
- ◯ No

5. If your answer was "No" to question nr 4, please specify the study population, and then go to the last question nr 8

- ◯ non-humans
- ◯ infants
- ◯ children
- ◯ adolescents

6. If your answer was "Yes" to question nr 2, is the publication type an original research article?

- ◯ Yes
- ◯ No

7. If your answer was "No" to question nr 6, please specify that the publication type is

- ◯ only abstract form
- ◯ conference proceeding
- ◯ letter
- ◯ review
- ◯ other

8. Have you answered "Yes" to all of the three questions: nr 2, 4, and 6?

- ◯ Yes
- ◯ No

Ta zawartość nie została stworzona ani zatwierdzona przez firmę Microsoft. Podane przez Ciebie informacje zostaną przesłane właścicielowi formularza.

Microsoft Forms

Technical aspects of inter-recti distance measurement with ultrasonographic imaging for physiotherapy purposes: The scoping review

Supplementary Material S3.

Publication Relevance Screening Form II

Publication relevance screening form: Full-text level

* Wymagane

* Ten formularz zarejestruje Twoje imię i nazwisko, dlatego wypełnij swoje imię i nazwisko.

1. Please, enter the publication title *

2. Is the publication related to physiotherapy or physical exercise/training fields? (please, give your answer based on screening the author's affiliation, study aims, and the main text)

   ◯ Yes

   ◯ No

3. If your answer was "No" to question nr 2, please, specify the field, and then go to question nr 5

   ◯ surgery (plastic/general)

   ◯ other field of medicine not related to physiotherapy, physical exercise/training

4. If your answer was "Yes" to question nr 2, please answer whether the publication includes the description of the ultrasound imaging (USI) measurement procedure of the inter-recti distance (IRD)/linea alba width/diastasis recti abdominis (DRA)

- ◯ Yes
- ◯ No

5. Have you answered "Yes" to both questions nr 2 and 4?

- ◯ Yes
- ◯ No

Ta zawartość nie została stworzona ani zatwierdzona przez firmę Microsoft. Podane przez Ciebie informacje zostaną przesłane właścicielowi formularza.

Microsoft Forms

Technical aspects of inter-recti distance measurement with ultrasonographic imaging for physiotherapy purposes: The scoping review

Supplementary Material S4.

Explanation and Elaboration Doc.

Explanation and elaboration:

Form I

Note: the database includes publications related to ophthalmology because muscles controlling the eyes are called recti muscles; therefore, they should not be confused with recti abdominis muscles

Reviewer's Decision:

- **if the reviewer has a problem answering any of the questions, the full text of the publication should be obtained for further appraisal and decision making at this phase.**
- **if the reviewer's answer is "Yes" to question nr 8, the publication will be included for further screening of full text at phase II.**

Form II

Reviewer's Decision:

- **if the reviewer's answer is "Yes" to question nr 5, the publication will be included in the scoping review**

Technical aspects of inter-recti distance measurement with ultrasonographic imaging for physiotherapy purposes: The scoping review

Supplementary Material S5.

Data Charting Form

Data charting form

Please, study the full text of the publication to copy and paste the following information:

First author<br>Publication year<br>Country or countries of the research center				
	**Publication nr/title**	**Reviewer 1**	**Reviewer 2**	**Final text abstraction (made jointly by two review leads)**
**1**				
**2**				
**3**				
**4**				
**5**				
**6**				
**7**				
**8**				
**9**				
**10**				
**11**				
**12**				
**13**				
**14**				
**15**				
**16**				
**17**				
**18**				
**19**				
**20**				
**21**				
**22**				

**23**				
**24**				
**25**				
**26**				
**27**				
**28**				
**29**				
**30**				
**31**				
**32**				
**33**				
**34**				
**35**				
**36**				
**37**				
**38**				
**39**				
**40**				

**Main study objective (please, copy and paste the relevant text from the abstract and the main text)**				
	**Publication nr/title**	**Reviewer 1**	**Reviewer 2**	**Final text abstraction (made jointly by two review leads)**
**1**				
**2**				
**3**				
**4**				
**5**				
**6**				
**7**				

8				
9				
10				
11				
12				
13				
14				
15				
16				
17				
18				
19				
20				
21				
22				
23				
24				
25				
26				
27				
28				
29				
30				
31				
32				
33				
34				
35				
36				
37				
38				

39				
40				

Population characteristics: sex, age and BMI (mean ± standard deviation); the information whether the subjects presented with DRA; in the case of women, please copy details, e.g., nulli-, primi-, multigravida/para, delivery: vaginal/cesarean				
	**Publication nr/title**	**Reviewer 1**	**Reviewer 2**	**Final text abstraction (made jointly by two review leads)**
1				
2				
3				
4				
5				
6				
7				
8				
9				
10				
11				
12				
13				
14				
15				
16				
17				
18				
19				
20				
21				
22				

**23**				
**24**				
**25**				
**26**				
**27**				
**28**				
**29**				
**30**				
**31**				
**32**				
**33**				
**34**				
**35**				
**36**				
**37**				
**38**				
**39**				
**40**				

**Description of the examinee's body position during an ultrasound assessment at rest (posture, position of the head and extremities)**				
	**Publication nr/title**	**Reviewer 1**	**Reviewer 2**	**Final text abstraction (made jointly by two review leads)**
**1**				
**2**				
**3**				
**4**				
**5**				
**6**				
**7**				

**8**				
**9**				
**10**				
**11**				
**12**				
**13**				
**14**				
**15**				
**16**				
**17**				
**18**				
**19**				
**20**				
**21**				
**22**				
**23**				
**24**				
**25**				
**26**				
**27**				
**28**				
**29**				
**30**				
**31**				
**32**				
**33**				
**34**				
**35**				
**36**				
**37**				
**38**				

**39**				
**40**				

**Description of the ultrasound assessment during specific muscle activity/task (the way of muscle activation during the examination)**				
	**Publication nr/title**	**Reviewer 1**	**Reviewer 2**	**Final text abstraction (made jointly by two review leads)**
**1**				
**2**				
**3**				
**4**				
**5**				
**6**				
**7**				
**8**				
**9**				
**10**				
**11**				
**12**				
**13**				
**14**				
**15**				
**16**				
**17**				
**18**				
**19**				
**20**				
**21**				
**22**				
**23**				

**24**				
**25**				
**26**				
**27**				
**28**				
**29**				
**30**				
**31**				
**32**				
**33**				
**34**				
**35**				
**36**				
**37**				
**38**				
**39**				
**40**				

**Breathing phase/pattern during image capturing**				
	**Publication nr/title**	**Reviewer 1**	**Reviewer 2**	**Final text abstraction (made jointly by two review leads)**
**1**				
**2**				
**3**				
**4**				
**5**				
**6**				
**7**				
**8**				

**9**				
**10**				
**11**				
**12**				
**13**				
**14**				
**15**				
**16**				
**17**				
**18**				
**19**				
**20**				
**21**				
**22**				
**23**				
**24**				
**25**				
**26**				
**27**				
**28**				
**29**				
**30**				
**31**				
**32**				
**33**				
**34**				
**35**				
**36**				
**37**				
**38**				
**39**				

**40**				

**Examiner's profession and experience (hours of training, years of practice)**				
	**Publication nr/title**	**Reviewer 1**	**Reviewer 2**	**Final text abstraction (made jointly by two review leads)**
**1**				
**2**				
**3**				
**4**				
**5**				
**6**				
**7**				
**8**				
**9**				
**10**				
**11**				
**12**				
**13**				
**14**				
**15**				
**16**				
**17**				
**18**				
**19**				
**20**				
**21**				
**22**				
**23**				
**24**				

**25**				
**26**				
**27**				
**28**				
**29**				
**30**				
**31**				
**32**				
**33**				
**34**				
**35**				
**36**				
**37**				
**38**				
**39**				
**40**				

**Ultrasound scanner (brand, type, mode, field of view) and transducer (type, frequency)**				
	**Publication nr/title**	**Reviewer 1**	**Reviewer 2**	**Final text abstraction (made jointly by two review leads)**
**1**				
**2**				
**3**				
**4**				
**5**				
**6**				
**7**				
**8**				
**9**				

**10**				
**11**				
**12**				
**13**				
**14**				
**15**				
**16**				
**17**				
**18**				
**19**				
**20**				
**21**				
**22**				
**23**				
**24**				
**25**				
**26**				
**27**				
**28**				
**29**				
**30**				
**31**				
**32**				
**33**				
**34**				
**35**				
**36**				
**37**				
**38**				
**39**				
**40**				

Choice of measurement site/sites along the linea alba				
	**Publication nr/title**	**Reviewer 1**	**Reviewer 2**	**Final text abstraction (made jointly by two review leads)**
**1**				
**2**				
**3**				
**4**				
**5**				
**6**				
**7**				
**8**				
**9**				
**10**				
**11**				
**12**				
**13**				
**14**				
**15**				
**16**				
**17**				
**18**				
**19**				
**20**				
**21**				
**22**				
**23**				
**24**				
**25**				

**26**				
**27**				
**28**				
**29**				
**30**				
**31**				
**32**				
**33**				
**34**				
**35**				
**36**				
**37**				
**38**				
**39**				
**40**				

**Use of cut-off values for "normal" inter-recti distance or diastasis recti abdominis (the values of the IRD considered to be normal or pathological condition)**				
	**Publication nr/title**	**Reviewer 1**	**Reviewer 2**	**Final text abstraction (made jointly by two review leads)**
**1**				
**2**				
**3**				
**4**				
**5**				
**6**				
**7**				
**8**				
**9**				

**10**				
**11**				
**12**				
**13**				
**14**				
**15**				
**16**				
**17**				
**18**				
**19**				
**20**				
**21**				
**22**				
**23**				
**24**				
**25**				
**26**				
**27**				
**28**				
**29**				
**30**				
**31**				
**32**				
**33**				
**34**				
**35**				
**36**				
**37**				
**38**				
**39**				
**40**				

Number of images which were taken at each measurement site and information whether the results of the IRD measurement were averaged				
	**Publication nr/title**	**Reviewer 1**	**Reviewer 2**	**Final text abstraction (made jointly by two review leads)**
**1**				
**2**				
**3**				
**4**				
**5**				
**6**				
**7**				
**8**				
**9**				
**10**				
**11**				
**12**				
**13**				
**14**				
**15**				
**16**				
**17**				
**18**				
**19**				
**20**				
**21**				
**22**				
**23**				
**24**				
**25**				

**26**				
**27**				
**28**				
**29**				
**30**				
**31**				
**32**				
**33**				
**34**				
**35**				
**36**				
**37**				
**38**				
**39**				
**40**				

**Description of the methods of the image processing and the inter-recti distance measurement (on-line or off-line)**				
	**Publication nr/title**	**Reviewer 1**	**Reviewer 2**	**Final text abstraction (made jointly by two review leads)**
**1**				
**2**				
**3**				
**4**				
**5**				
**6**				
**7**				
**8**				
**9**				
**10**				

**11**				
**12**				
**13**				
**14**				
**15**				
**16**				
**17**				
**18**				
**19**				
**20**				
**21**				
**22**				
**23**				
**24**				
**25**				
**26**				
**27**				
**28**				
**29**				
**30**				
**31**				
**32**				
**33**				
**34**				
**35**				
**36**				
**37**				
**38**				
**39**				
**40**				

	**Publication nr/title**	**Reviewer 1**	**Reviewer 2**	**Final text abstraction (made jointly by two review leads)**
**1**				
**2**				
**3**				
**4**				
**5**				
**6**				
**7**				
**8**				
**9**				
**10**				
**11**				
**12**				
**13**				
**14**				
**15**				
**16**				
**17**				
**18**				
**19**				
**20**				
**21**				
**22**				
**23**				
**24**				
**25**				
**26**				

**27**				
**28**				
**29**				
**30**				
**31**				
**32**				
**33**				
**34**				
**35**				
**36**				
**37**				
**38**				
**39**				
**40**				

Technical aspects of inter-recti distance measurement with ultrasonographic imaging for physiotherapy purposes: The scoping review

Supplementary Material S6. Objectives of the Studies

In the 49 reviewed studies, the main objectives were to investigate/assess the following:

- the reliability of IRD measurements using USI (intrarater and/or interrater reliability) [44-47, 51, 52, 60];
- the validity, accuracy, and agreement of IRD measurements using a caliper [57-59, 64], tape measure [64], finger width [64, 68], or USI with an extended field of view [51] compared with the conventional USI;
- the impact of a specific task (abdominal or pelvic floor muscle activation [8, 9, 11, 13, 32-35, 55, 61, 70, 71], abdominal muscle activation with Tubigrip or taping [61]), or posture [60] on IRD, and the impact of specific tasks on IRD, linea alba stiffness [8], distortion [8, 32, 71] and abdominal muscle thickness [55, 71];
- the effect of a specific measurement site [8, 60] and the transducer tilt [50] on IRD and linea alba stiffness and distortion [8];
- changes in IRD, rectus abdominis size and shape, and abdominal muscle strength in postpartum women over time [16, 18];
- IRD/DRA in relation to symptom severity [20], physical and psychological factors [65], abdominal/trunk muscle function [17, 18], lumbopelvic pain [15], or pelvic floor trauma [27] in postpartum women;
- IRD, abdominal muscles, and fasciae in paras depending on the delivery mode [12, 70]; the differences in IRD and abdominopelvic function between nulliparas, primiparas, and multiparas [53], the differences in IRD, abdominal muscle thickness, and sheer wave speed between DRA and non-DRA subjects [62];

- “normal” IRD width in primiparas during pregnancy and postpartum [69], DRA prevalence and risk factors in pregnant/postpartum women [15], and the anatomical variations of DRA [10];
- the impacts of abdominal binding and trunk exercises [31], progressive yoga exercises [63], core stabilization exercise program (conducted in person and online) [66] on DRA/IRD and abdominal muscle thickness [66] in postpartum women; the effects of a specific exercise program carried out in pregnancy on pregnancy and postpartum IRD [72], and the effects of different physical therapy programs on the rectus abdominis thickness and IRD in patients with Achilles tendinopathy [56] in intervention studies;
- IRD, abdominal muscle and perimuscular connective tissue thickness in patients with Achilles tendinopathy [41], primary dysmenorrhea [42], and lumbopelvic pain [43], and in athletes [39, 40]; IRD, anteroposterior diameter of the levator ani hiatus, and abdominal muscle thickness in women with dyspareunia [54]; IRD, pubic symphysis separation, and pain catastrophizing in women with pelvic girdle pain [67] (Table 2).
